# Renal proximal tubular NEMO plays a critical role in ischemic acute kidney injury

**DOI:** 10.1172/jci.insight.139246

**Published:** 2020-09-17

**Authors:** Sang Jun Han, Ryan M. Williams, Mihwa Kim, Daniel A. Heller, Vivette D’Agati, Marc Schmidt-Supprian, H. Thomas Lee

**Affiliations:** 1 Department of Anesthesiology, College of Physicians and Surgeons of Columbia University, New York, New York, USA.; 2 Department of Biomedical Engineering, City College of New York, New York, New York, USA.; 3 Department of Molecular Pharmacology & Chemistry, Memorial Sloan Kettering Cancer Center, New York, New York, USA.; 4 Department of Pathology, College of Physicians and Surgeons of Columbia University, New York, New York, USA.; 5 Institute of Experimental Hematology, School of Medicine, Technical University Munich, Munich, Germany.

**Keywords:** Inflammation, Pharmacology

## Abstract

We determined that renal proximal tubular (PT) NF-κB essential modulator (NEMO) plays a direct and critical role in ischemic acute kidney injury (AKI) using mice lacking renal PT NEMO and by targeted renal PT NEMO inhibition with mesoscale nanoparticle–encapsulated NEMO binding peptide (NBP MNP). We subjected renal PT NEMO–deficient mice, WT mice, and C57BL/6 mice to sham surgery or 30 minutes of renal ischemia and reperfusion (IR). C57BL/6 mice received NBP MNP or empty MNP before renal IR injury. Mice treated with NBP MNP and mice deficient in renal PT NEMO were protected against ischemic AKI, having decreased renal tubular necrosis, inflammation, and apoptosis compared with control MNP-treated or WT mice, respectively. Recombinant peptidylarginine deiminase type 4 (rPAD4) targeted kidney PT NEMO to exacerbate ischemic AKI in that exogenous rPAD4 exacerbated renal IR injury in WT mice but not in renal PT NEMO–deficient mice. Furthermore, rPAD4 upregulated proinflammatory cytokine mRNA and NF-κB activation in freshly isolated renal proximal tubules from WT mice but not from PT NEMO–deficient mice. Taken together, our studies suggest that renal PT NEMO plays a critical role in ischemic AKI by promoting renal tubular inflammation, apoptosis, and necrosis.

## Abstract 2

Renal proximal tubular NFκB essential modulator (NEMO) regulates ischemic acute kidney injury by promoting renal tubular inflammation, apoptosis, and necrosis.

## Introduction

Acute kidney injury (AKI) is a major clinical problem, but there is no effective therapy (1–3). Renal ischemia and reperfusion (IR) injury is a leading cause of AKI, as patients undergoing cardiac, vascular, or liver transplant surgical procedures have approximately a 50%–80% chance of developing ischemic AKI (4, 5). Renal IR results in rapid proximal tubular necrosis followed by tubular inflammatory response with leukocyte influx (6–8). Indeed, upregulation of renal tubular proinflammatory cytokines and chemokines causes influx of inflammatory leukocytes into the renal parenchyma and exacerbates ischemic AKI (9–11). Therefore, regulating the renal tubular inflammatory process after renal IR could potentially lead to therapy to protect against ischemic AKI.

We previously showed that renal tubular peptidylarginine deiminase type 4 (PAD4), a calcium-dependent enzyme that catalyzes the conversion of peptidylarginine residues to peptidylcitrulline, is rapidly upregulated after renal IR injury (12, 13). Furthermore, we demonstrated that kidney PAD4 plays a critical role in ischemic AKI in that PAD4 inhibitors or genetic deletion of PAD4 protected against ischemic AKI (12). We also showed that recombinant PAD4 (rPAD4) worsens ischemic AKI by promoting renal tubular inflammation via NF-κB activation, renal tubular cytokine generation, and neutrophil infiltration (12). Renal tubular PAD4 induction is mediated by P2X7 receptor activation secondary to release of ATP from necrotic renal cells (14). Finally, we showed that renal tubular PAD4 promotes a proinflammatory arm of the NF-κB activation pathway by selectively citrullinating IKKγ (NF-κB essential modulator [NEMO] — a regulatory subunit of the IKK complex) over IKKα or IKKβ subtypes. NEMO inhibition with NEMO binding peptide (NBP) attenuated renal tubular NF-κB activation, proinflammatory gene induction, renal tubular neutrophil infiltration, and ischemic AKI in mice (15).

However, since NEMO is present in every cell type in the kidney as well as extrarenal organs, it remains to be determined whether renal PT NEMO plays a critical role in generating ischemic AKI by PAD4-mediated citrullination. Furthermore, since NEMO plays important and diverse physiological roles in almost all cell types, systemic NEMO inhibition is not feasible as a therapeutic option to treat ischemic AKI. Therefore, in this study, we tested whether renal PT NEMO plays a critical role in ischemic AKI using 2 distinct mechanistic approaches. We generated and subjected mice with renal PT NEMO deficiency to renal IR injury. We also used a potentially novel mesoscale nanoparticle–mediated (MNP-mediated) drug delivery approach to selectively deliver NBP to renal proximal tubular cells to protect against ischemic AKI.

## Results

### Confirmation of renal PT NEMO deletion in NEMO^fl/fl^ PEPCK-Cre mice.

**Figure 1:**
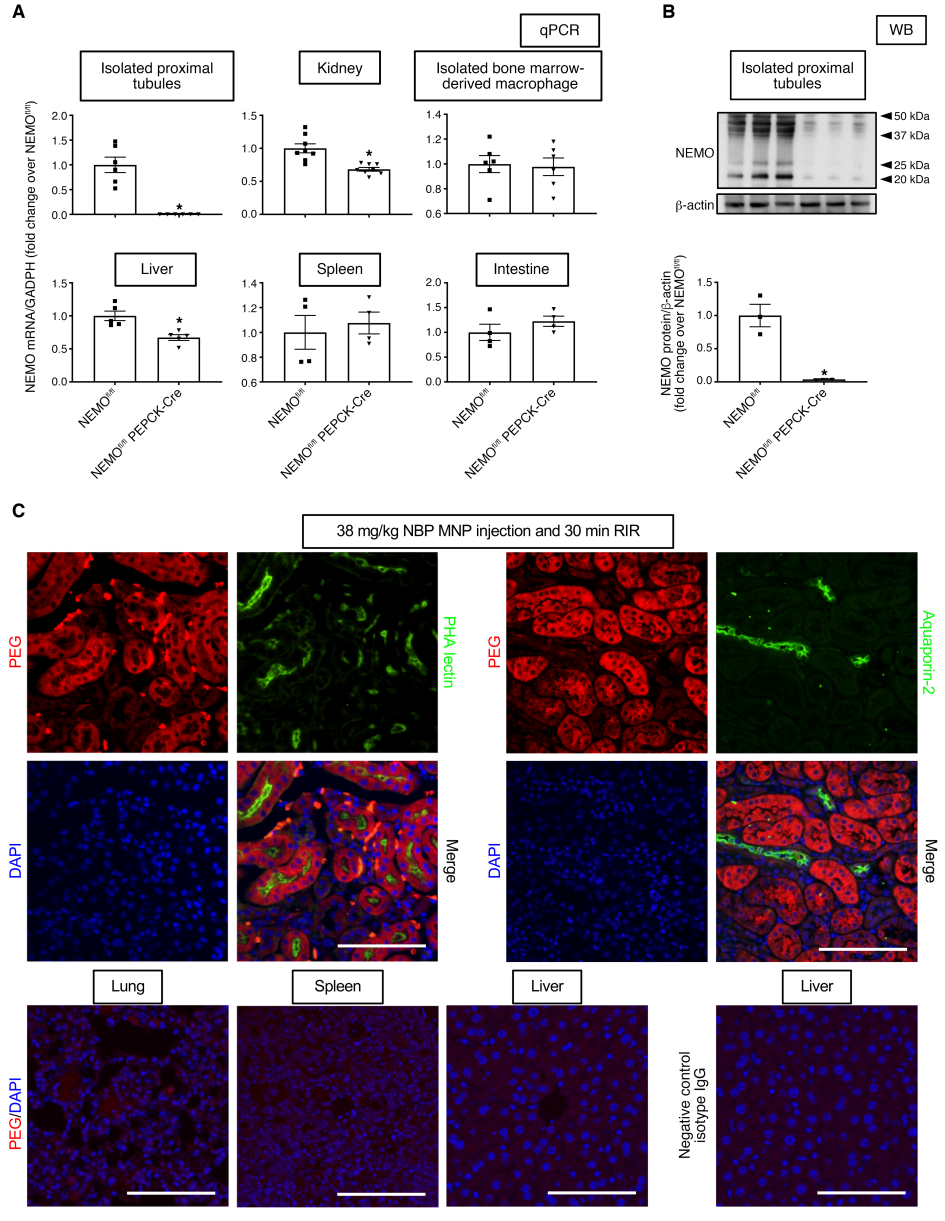
Confirmation of renal proximal tubule cell NEMO deletion in proximal tubule cell NEMO–null mice. (**A**) NEMO mRNA normalized to GAPDH from qRT-PCR reactions in isolated proximal tubule cells, bone marrow–derived macrophages, whole kidney, liver, spleen, and small intestine of WT NEMO^fl/fl^ and NEMO^fl/fl^ PEPCK-Cre mice. (**B**) NEMO protein normalized to β-actin in isolated proximal tubule cells of NEMO^fl/fl^ and NEMO^fl/fl^ PEPCK-Cre mice. The bands below approximately 50 kDA predicted molecular weight of NEMO may represent truncated forms, varying glycosylation products, and cleaved fragments. For statistical analysis, Student’s *t* test was used to detect significant changes. *P < 0.05 versus NEMO^fl/fl^ mice (*N* = 3–8). Error bars represent 1 SEM. (**C**) Confirmation of renal proximal tubule cell–specific MNP delivery. Mouse kidney sections stained with anti-PEG antibody to detect MNP localization and PHA-lectin to mark renal proximal tubule cells or with anti–aquaporin-2 antibody to mark renal collecting ducts and counterstained with DAPI to visualize cell nuclei. Mice were injected with 38 mg/kg NBP MNP or with vehicle control 6 hours before renal IR injury. Renal proximal tubular distribution pattern for PEG was confirmed by colocalization of PEG antibody staining and PHA lectin staining in MNP-injected mice (×200 original magnification images shown, representative of 3 experiments). However, PEG staining was not detected in the lung, spleen, or liver (×200 original magnification images shown, representative of 3 experiments). Scale bar: 50 μm.

NEMO mRNA and protein expression in renal proximal tubules from NEMO^fl/fl^ phosphoenolpyruvate carboxykinase promoter–Cre (PEPCK-Cre) mice was decreased by more than 99.99% and more than 96%, respectively, compared with NEMO^fl/fl^ mice (Figure 1, A and B). However, NEMO mRNA expression in isolated bone marrow cells, intestine, and spleen was equivalent between NEMO^fl/fl^ PEPCK-Cre mice and NEMO^fl/fl^ mice. Whole kidney and liver NEMO mRNA expressions were approximately 32% lower in NEMO^fl/fl^ PEPCK-Cre mice compared with NEMO^fl/fl^ mice (Figure 1A). These findings are consistent with renal proximal tubular and periportal hepatocyte expression of PEPCK-Cre recombinase (16, 17).

### Generation of MNPs containing NBP and confirmation of renal tubular MNP delivery.

Lyophilized MNPs containing NBP exhibited a mean hydrodynamic diameter of 315.4 ± 4.9 nm and a polydispersity index (PDI) of 0.29 ± 0.03. These particles encapsulated 431 ng NBP/1 mg MNP for an encapsulation efficiency of 89%. Empty control MNPs were 332.0 ± 7.6 nm in diameter with a PDI of 0.30 ± 0.04. The renal proximal tubular distribution pattern for the MNPs was confirmed by colocalization of PEG staining with phytohemagglutinin (PHA) lectin staining in mice injected with 38 mg/kg MNP (Figure 1C, representative of 3 experiments). PEG staining did not colocalize with the collecting duct–specific AQP-2 staining, and PEG staining was not detected in other tissues, including the lung, spleen, and liver. These results demonstrated preferential localization of MNPs in renal proximal tubular cells.

### Kidney PT NEMO deletion or selective tubular delivery of NBP encapsulated in MNP protects against ischemic AKI in mice.

**Figure 2:**
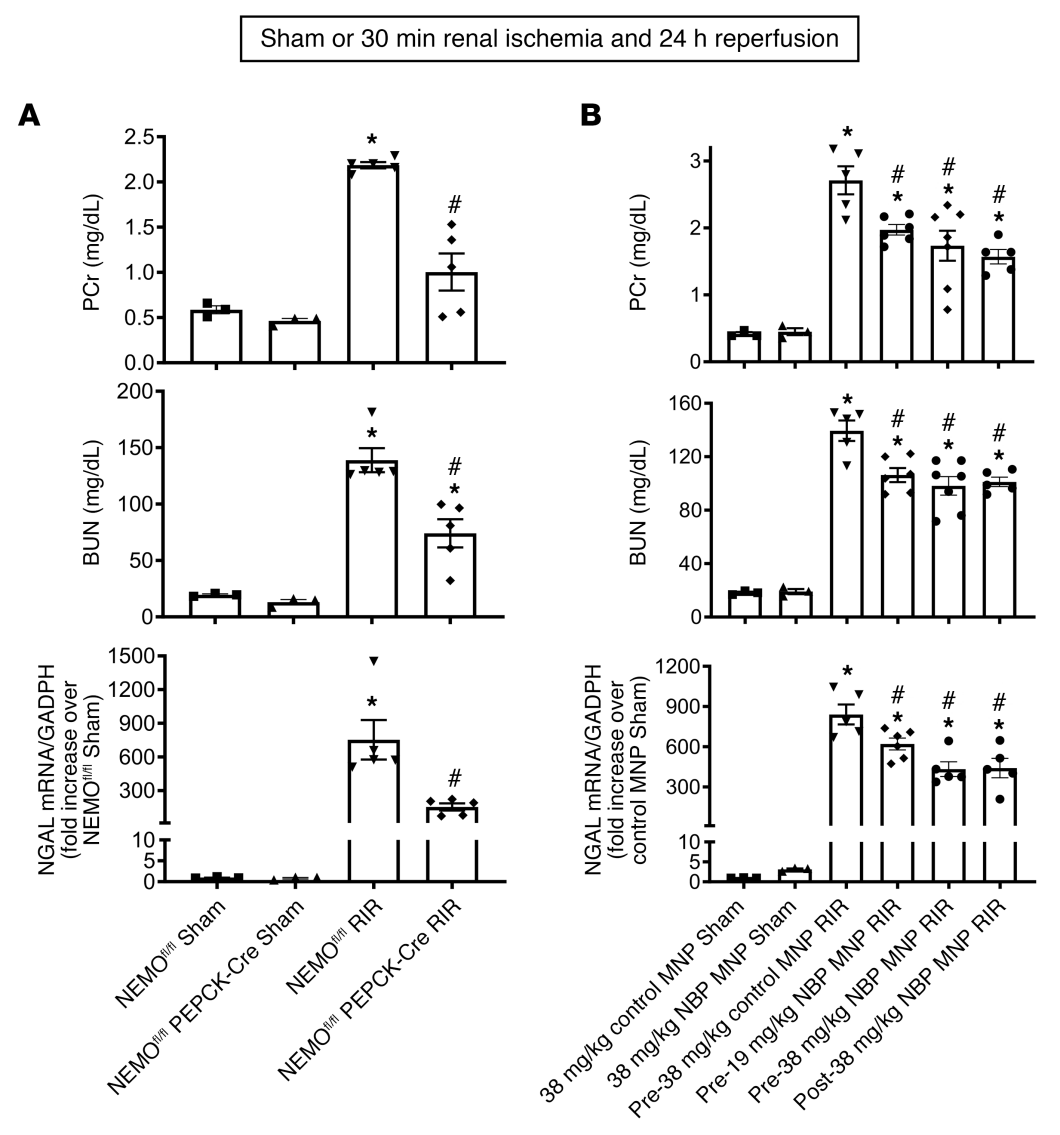
Kidney PT NEMO deletion or selective tubular delivery of NBP MNP protects against ischemic AKI in mice. (**A**) NEMO^fl/fl^ WT mice or renal PT NEMO–deficient (NEMO^fl/fl^ PEPCK-Cre) mice were subjected to sham surgery (*N* = 3) or to 30 minutes renal IR (*N* = 5). (**B**) Separate cohorts of C57BL/6 mice were injected with control MNP or with 19 or 38 mg/kg NBP encapsulated in MNPs (NBP MNP) 6 hours before sham surgery (*N* = 3) or 30 minutes renal ischemia (IR, *N* = 5–7). Some mice were injected with 38 mg/kg NBP MNP 15 minutes after reperfusion (IR, *N* = 5). Twenty-four hours later, plasma BUN and creatinine as well as kidney NGAL mRNA were measured. For statistical analysis, 1-way ANOVA plus Tukey’s post hoc multiple-comparisons test was used to detect significant changes. *P < 0.05 versus WT mice or control MNP-injected mice subjected to sham surgery. ^#^*P* < 0.05 versus WT mice or control MNP-injected mice subjected to renal IR. Error bars represent 1 SEM.

Plasma creatinine and blood urea nitrogen (BUN) values were similar between NEMO^fl/fl^ mice and renal PT NEMO–null mice subjected to a sham operation (Figure 2A). NEMO^fl/fl^ mice subjected to renal IR had significantly higher plasma creatinine and BUN as well as kidney neutrophil gelatinase-associated lipocalin (NGAL) mRNA (*N* = 5) compared with sham-operated NEMO^fl/fl^ mice (*N* = 3). In preliminary studies, we determined that PEPCK-Cre mice subjected to renal IR had a similar degree of renal injury when compared with NEMO^fl/fl^ mice (data not shown). We found here that renal PT NEMO–null mice subjected to renal IR were protected against ischemic AKI compared with control NEMO^fl/fl^ mice, as demonstrated by reduced plasma creatinine and BUN as well as kidney NGAL mRNA expression (*N* = 5).

Since liver NEMO mRNA expressions were approximately 32% lower in NEMO^fl/fl^ PEPCK-Cre mice compared with NEMO^fl/fl^ mice given that periportal hepatocytes express PEPCK-Cre recombinase (16, 17), we determined whether there are differences in hepatic injury after ischemic AKI between NEMO^fl/fl^ PEPCK-Cre mice and NEMO^fl/fl^ mice. There were no differences in alanine aminotransferase (ALT) levels between sham-operated NEMO^fl/fl^ mice (18 ± 10 U/L, *N* = 3) and NEMO^fl/fl^ PEPCK-Cre mice (27 ± 6 U/L, *N* = 3). Moreover, there were no differences in ALT levels between NEMO^fl/fl^ mice (146 ± 19 U/L, *N* = 5) and NEMO^fl/fl^ PEPCK-Cre mice (145 ± 23 U/L, *N* = 5) subjected to renal IR. In addition, histological liver injuries were similar after renal IR between NEMO^fl/fl^ mice and NEMO^fl/fl^ PEPCK-Cre mice (Supplemental Figure 1; supplemental material available online with this article; https://doi.org/10.1172/jci.insight.139246DS1).

Plasma creatinine and BUN values were similar between sham-operated C57BL/6 mice injected with empty control MNPs and 38 mg/kg NBP encapsulated in MNPs (NBP MNP, *N* = 3, Figure 2B). Mice treated with control MNPs and subjected to renal IR had significantly higher plasma creatinine and BUN as well as kidney NGAL mRNA (*N* = 5) compared with sham-operated mice (*N* = 3). We showed here that mice treated with 19 or 38 mg/kg NBP MNP 6 hours before renal ischemia or mice treated with 38 mg/kg NBP MNP 15 minutes after reperfusion were protected against ischemic AKI compared with control MNP-treated mice, as demonstrated by reduced plasma creatinine and BUN as well as kidney NGAL mRNA expression (*N* = 5–7).

### Kidney PT NEMO deletion or selective tubular delivery of NBP MNP reduces renal tubular necrosis after ischemic AKI.

**Figure 3:**
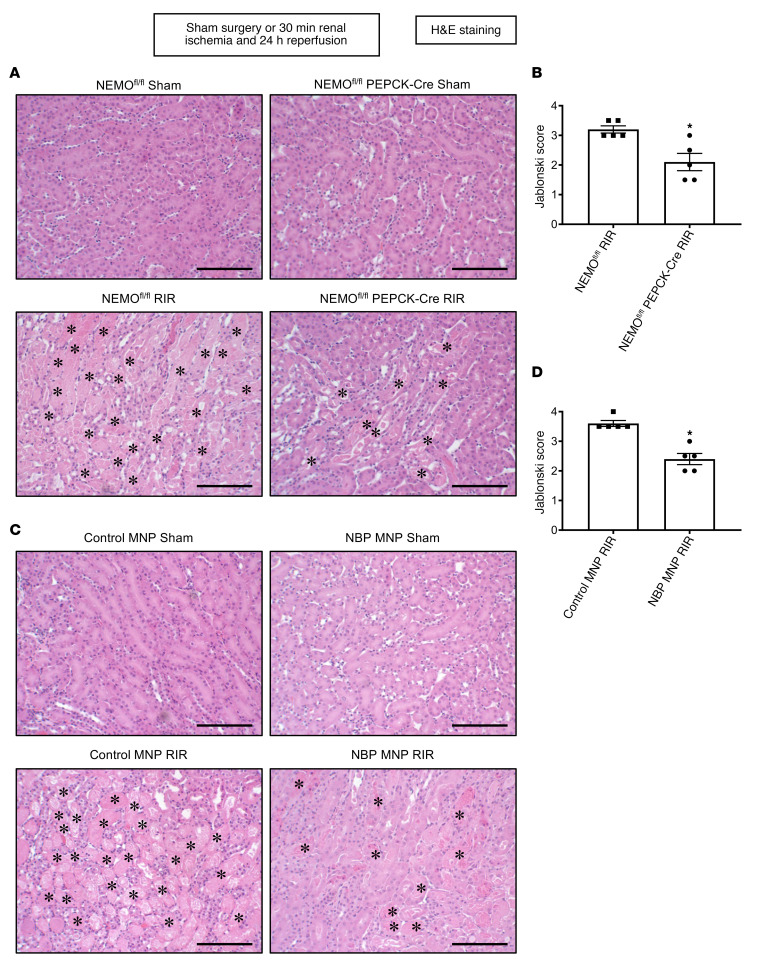
Kidney PT NEMO deletion or selective tubular delivery of NBP MNP reduces renal tubular necrosis after ischemic AKI. Representative H&E images (×200 original magnification, **A** and **C**) and Jablonski renal injury scores assessing the degree of renal tubular necrosis (scale: 0–4, **B** and **D**) of kidneys of mice subjected to sham surgery or to 30 minutes renal ischemia and 24 hours reperfusion (×200 original magnification). (**A** and **B**) NEMO^fl/fl^ WT mice or renal PT NEMO–deficient mice subjected to sham surgery or renal IR (*N* = 5). (**C** and **D**) C57BL/6 mice injected with control MNP or with 19 or 38 mg/kg NBP MNP 6 hours before sham surgery or renal IR (*N* = 5). Severe tubular necrosis and proteinaceous casts (*) are indicated in H&E images. Scale bar: 200 μm. *P < 0.05 versus WT mice or control MNP-injected mice subjected to renal IR. Error bars represent 1 SEM. For statistical analysis, the Mann-Whitney nonparametric test was used to detect significant changes.

Figure 3A shows representative kidney H&E images of NEMO^fl/fl^ mice and renal PT NEMO–null mice subjected to sham surgery or 30 minutes of renal IR and 24 hours of reperfusion (original magnification, ×200, *N* = 5). NEMO^fl/fl^ mice subjected to renal IR showed severe tubular necrosis and proteinaceous casts as well as increased tubular dilatation and congestion. In contrast, renal PT NEMO–null mice had decreased renal tubular necrosis, congestion, and cast formation compared with NEMO^fl/fl^ mice subjected to renal IR. Kidneys from renal PT NEMO–null mice had significantly reduced renal tubular injury scores compared with control MNP-injected mice after IR (Figure 3B).

Figure 3C shows representative kidney H&E images of control MNP-injected or 38 mg/kg NBP MNP–injected mice subjected to sham surgery or 30 minutes of renal IR and 24 hours of reperfusion (original magnification, ×200, *N* = 5). Control MNP-injected mice subjected to renal IR showed severe tubular necrosis and proteinaceous casts as well as increased tubular dilatation and congestion. Treatment with 38 mg/kg NBP MNP 6 hours before renal ischemia decreased renal tubular necrosis, congestion, and cast formation compared with the control MNP-injected mice subjected to renal IR. Kidneys from NBP MNP–injected mice had significantly reduced renal tubular injury scores compared with control MNP-injected mice after IR (Figure 3D).

### Kidney PT NEMO deletion or selective tubular delivery of NBP MNP attenuates kidney apoptosis in mice after ischemic AKI.

**Figure 4:**
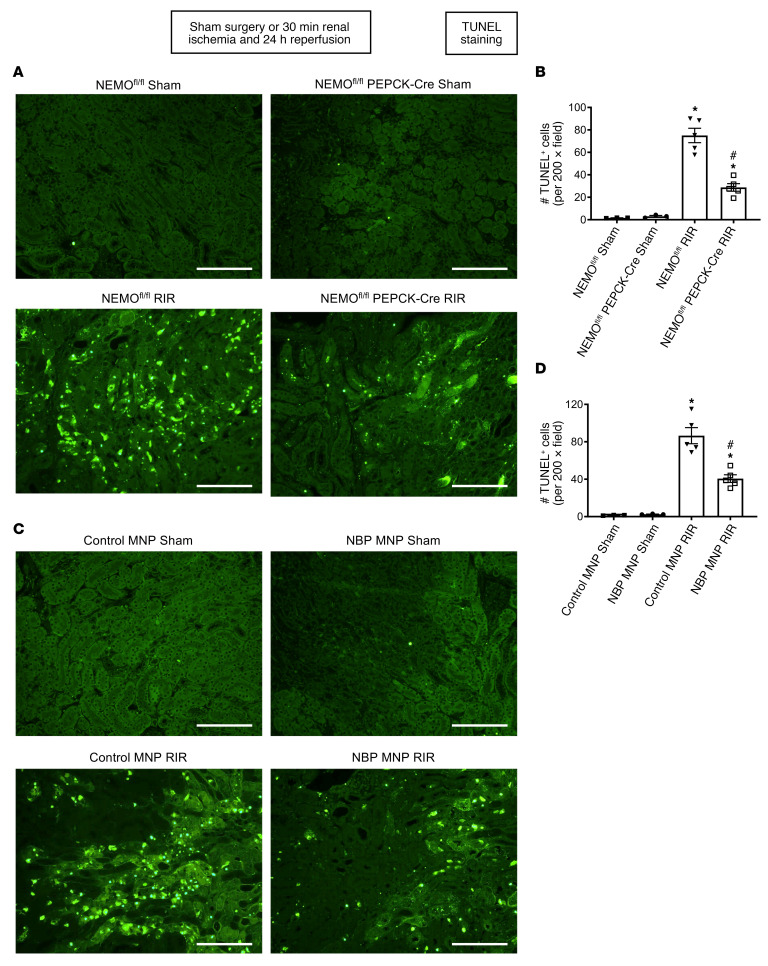
Kidney PT NEMO deletion or selective tubular delivery of NBP MNP attenuates kidney apoptosis after ischemic AKI. Representative images of TUNEL staining (**A** and **C**) indicative of renal tubular apoptosis and counts of TUNEL-positive kidney cells (**B** and **D**) in the kidneys of mice subjected to sham surgery (*N* = 3) or to 30 minutes renal ischemia and 24 hours reperfusion (*N* = 5, ×200 original magnification). (**A** and **B**) NEMO^fl/fl^ WT mice or renal PT NEMO–deficient mice subjected to sham surgery or renal IR. (**C** and **D**) C57BL/6 mice injected with control MNP or with 38 mg/kg NBP MNP 6 hours before sham surgery (*N* = 3) or renal IR (*N* = 5). Scale bar: 200 μm. For statistical analysis, 1-way ANOVA plus Tukey’s post hoc multiple-comparisons test was used to detect significant changes. *P < 0.05 versus WT mice or control MNP-injected mice subjected to sham surgery. ^#^*P* < 0.05 versus WT mice or control MNP-injected mice subjected to renal IR. Error bars represent 1 SEM.

Figure 4A shows representative TUNEL-stained images indicative of renal apoptosis and counts of TUNEL-positive kidney cells (Figure 4B) from NEMO^fl/fl^ mice and renal PT NEMO–null mice subjected to sham surgery (*N* = 3) or to renal IR (*N* = 5, original magnification, ×200). We detected many positive TUNEL (fragmented DNA) cells, suggestive of renal tubular apoptosis in the kidneys from NEMO^fl/fl^ mice subjected to renal IR injury. TUNEL-positive kidney cell counts were significantly reduced in renal PT NEMO–null mice subjected to renal IR injury.

Figure 4C shows representative TUNEL-stained images indicative of renal apoptosis and counts of TUNEL-positive kidney cells (Figure 4D) from control MNP-injected and 38 mg/kg NBP MNP–injected mice subjected to sham surgery (*N* = 3) or to renal IR (*N* = 5, original magnification, ×200). We detected many positive TUNEL (fragmented DNA) cells, suggestive of renal tubular apoptosis in the kidneys from control MNP-injected mice subjected to renal IR injury. TUNEL-positive kidney cell counts were significantly reduced in NBP MNP–injected mice compared with control MNP-injected mice after IR.

### Kidney PT NEMO deletion or selective tubular delivery of NBP MNPs reduces kidney neutrophil infiltration after ischemic AKI.

**Figure 5:**
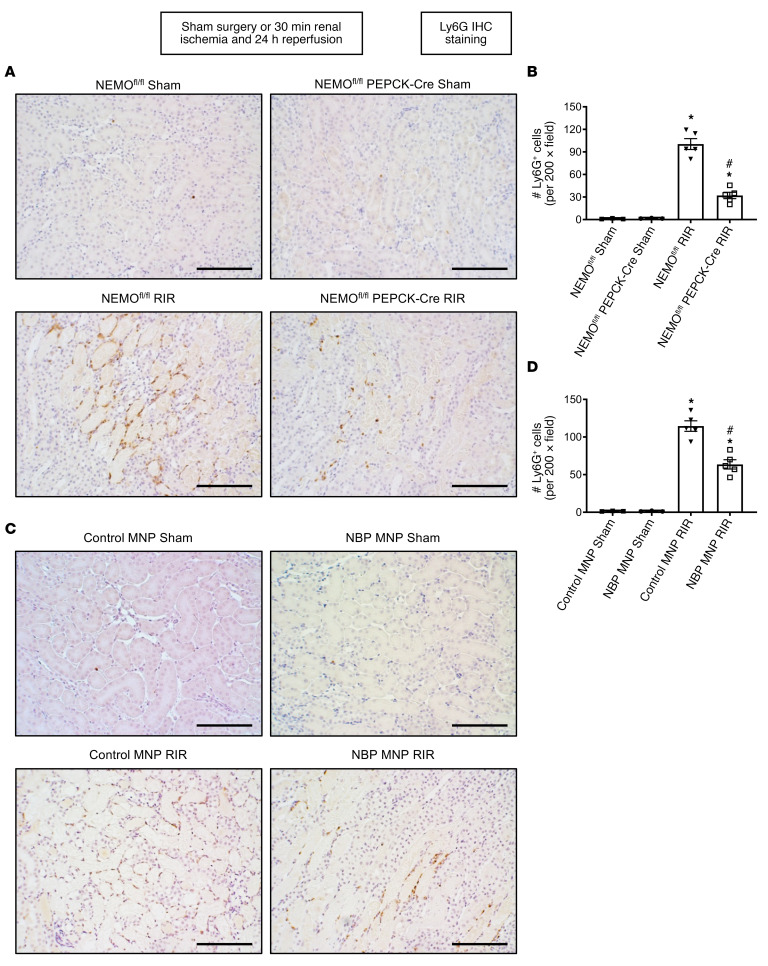
Kidney PT NEMO deletion or selective tubular delivery of NBP MNP decreases kidney neutrophil infiltration after ischemic AKI. (**A**) Representative images of IHC for neutrophils (dark brown, **A** and **C**) and counts of infiltrating kidney neutrophils (**B** and **D**) in the kidneys of mice subjected to sham surgery (*N* = 3) or to 30 minutes of renal ischemia and 24 hours of reperfusion (*N* = 5, ×200 original magnification). (**A** and **B**) NEMO^fl/fl^ WT mice or renal PT NEMO–deficient mice subjected to sham surgery or renal IR. (**C** and **D**) C57BL/6 mice injected with control MNP or with 38 mg/kg NBP MNP 6 hours before sham surgery (*N* = 3) or renal IR (*N* = 5). Scale bar: 200 μm. For statistical analysis, 1-way ANOVA plus Tukey’s post hoc multiple-comparisons test was used to detect significant changes. *P < 0.05 versus WT mice or control MNP-injected mice subjected to sham surgery. ^#^*P* < 0.05 versus WT mice or control MNP-injected mice subjected to renal IR. Error bars represent 1 SEM.

Figure 5A shows representative IHC images and counts of infiltrating kidney neutrophils in the kidneys (Figure 5B) of NEMO^fl/fl^ mice and renal PT NEMO–null mice subjected to sham surgery (*N* = 3) or renal IR (*N* = 5, original magnification, ×200). Kidney neutrophil infiltration increased significantly in NEMO^fl/fl^ mice subjected to renal IR. Neutrophil infiltration was significantly reduced in renal PT NEMO–null mice subjected to renal IR injury.

Figure 5C shows representative IHC image counts of infiltrating kidney neutrophils in the kidneys (Figure 5D) of control MNP-injected mice and 38 mg/kg NBP MNP–injected mice subjected to sham surgery (*N* = 3) or renal IR (*N* = 5, original magnification, ×200). Kidney neutrophil infiltration was significantly higher in control MNP-injected mice subjected to renal IR. NBP MNP treatment significantly attenuated kidney neutrophil infiltration after renal IR compared with NBP MNP–injected mice.

### Kidney PT NEMO deletion or selective tubular delivery of NBP MNPs downregulates proinflammatory chemokine and cytokine induction and renal tubular NF-κB activation after ischemic AKI.

**Figure 6:**
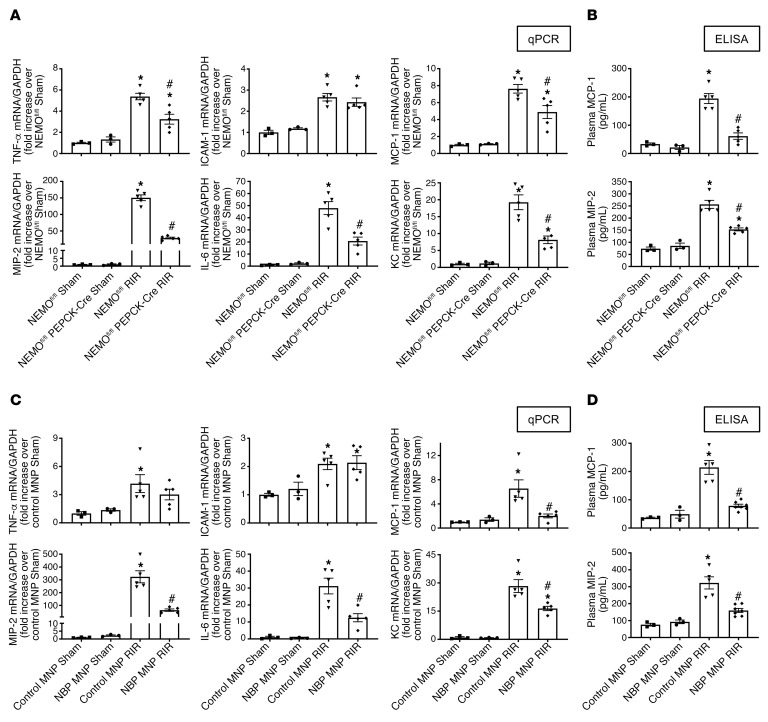
Kidney PT NEMO deletion or selective tubular delivery of NBP MNP attenuates kidney proinflammatory chemokine/cytokine induction after ischemic AKI. With quantitative RT-PCR (**A** and **C**), we measured the expression of proinflammatory cytokine and chemokine mRNAs in the kidney (KC, MCP-1, MIP-2, TNF-α, IL-6, and ICAM-1), and with ELISA (**B** and **D**), we measured the plasma MCP-1 and MIP-2 levels 24 hours after sham surgery or 30 minutes after renal ischemia. (**A** and **B**) NEMO^fl/fl^ WT mice or renal PT NEMO–deficient mice subjected to sham surgery or renal IR. (**C** and **D**) C57BL/6 mice injected with control MNP or with 38 mg/kg NBP MNP 6 hours before sham surgery (*N* = 3) or renal IR (*N* = 5). Fold increases in proinflammatory mRNAs normalized to GAPDH from quantitative RT-PCR reactions for each indicated mRNA are shown. For statistical analysis, 1-way ANOVA plus Tukey’s post hoc multiple-comparisons test was used to detect significant changes. *P < 0.05 versus WT mice or control MNP-injected mice subjected to sham surgery. ^#^*P* < 0.05 versus WT mice or control MNP-injected mice subjected to renal IR. Error bars represent 1 SEM. KC, keratinocyte-derived cytokine; MCP-1, monocyte chemoattractive protein-1; MIP-2, macrophage inflammatory protein-2.

**Figure 7:**
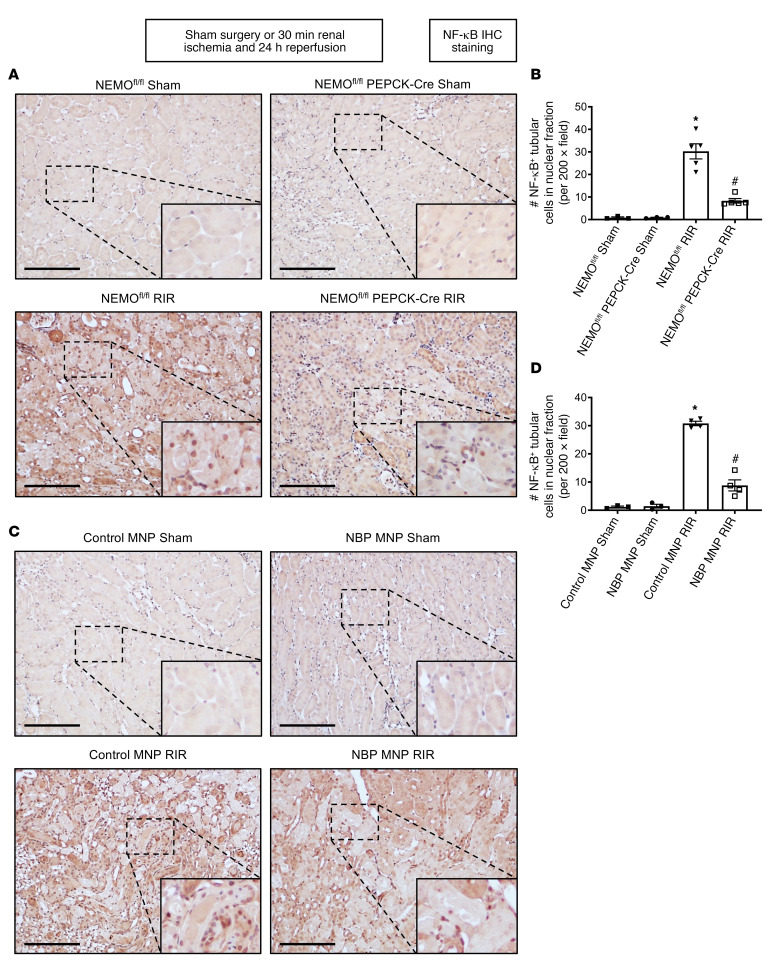
Kidney PT NEMO deletion or selective tubular delivery of NBP MNP attenuates renal tubular NF-κB activation after ischemic AKI. Representative images of IHC for NF-κB (**A** and **C**) and counts of NF-κB–positive tubular cells in nuclear fraction (**B** and **D**) in the kidneys of mice subjected to sham surgery (*N* = 3) or to 30 minutes of renal ischemia and 24 hours of reperfusion (*N* = 5, ×200 original magnification). Scale bar: 200 μm. For statistical analysis, 1-way ANOVA plus Tukey’s post hoc multiple-comparisons test was used to detect significant changes. *P < 0.05 versus WT mice or control MNP-injected mice subjected to sham surgery. ^#^*P* < 0.05 versus WT mice or control MNP-injected mice subjected to renal IR. Error bars represent 1 SEM.

**Figure 8:**
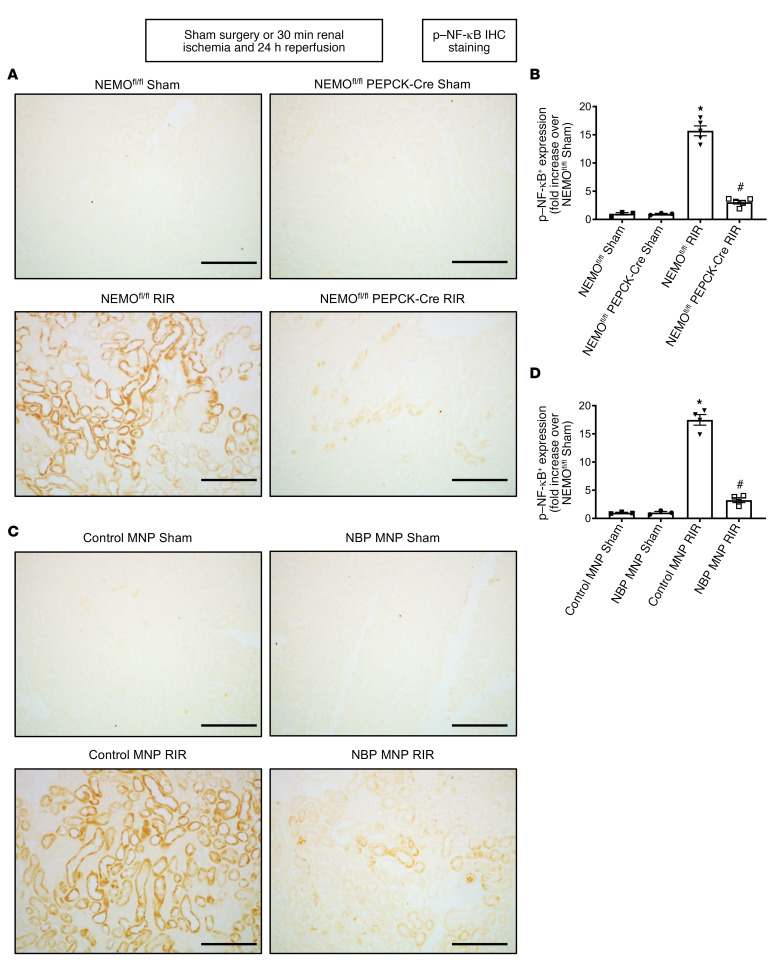
Kidney PT NEMO deletion or selective tubular delivery of NBP MNP attenuates renal tubular phospho–NF-κB activation after ischemic AKI. Representative images of IHC for phosphorylated NF-κB (p–NF-κB, **A** and **C**) and counts of p–NF-κB (**B** and **D**) in the kidneys of mice subjected to sham surgery (*N* = 3) or to 30 minutes of renal ischemia and 24 hours of reperfusion (*N* = 5, ×200 original magnification). Scale bar: 200 μm. For statistical analysis, 1-way ANOVA plus Tukey’s post hoc multiple-comparisons test was used to detect significant changes. *P < 0.05 versus WT mice or control MNP-injected mice subjected to sham surgery. ^#^*P* < 0.05 versus WT mice or control MNP-injected mice subjected to renal IR. Error bars represent 1 SEM.

Figure 6A shows fold increases in proinflammatory mRNAs normalized to GAPDH for each indicated mRNA in the kidneys of NEMO^fl/fl^ mice and renal PT NEMO–null mice subjected to sham surgery (*N* = 3) or renal IR (*N* = 5). Figure 6C shows fold increases in proinflammatory mRNAs normalized to GAPDH for each indicated mRNA in the kidneys of control MNP-injected mice and 38 mg/kg NBP MNP–injected mice subjected to sham surgery (*N* = 3) or renal IR (*N* = 5). Ischemic AKI increased all proinflammatory genes measured in NEMO^fl/fl^ control mice or control MNP-injected mice. Consistent with the renal protective role of NEMO inhibition or deletion via reduction of neutrophil- and macrophage-attracting chemokines, we showed that macrophage inflammatory protein-2 (MIP-2) and monocyte chemoattractive protein-1 (MCP-1) mRNA expressions as well as plasma MIP-2 and MCP-1 protein levels (Figure 6, B and D) were significantly attenuated in renal PT NEMO–null mice or NBP MNP–injected mice. Moreover, IL-6, TNF-α, and keratinocyte chemoattractant (KC) induction were attenuated in NBP MNP–injected mice or mice deficient in renal PT NEMO. Finally, renal IR significantly increased expressions of NF-κB and phosphorylated NF-κB (p-NF-κB) as well as NF-κB nuclear translocation into renal tubule cells. Kidney PT NEMO deletion or selective tubular delivery of NBP MNP treatment attenuated these increases in NF-κB and p-NF-κB expression (Figure 7 and Figure 8).

### PAD4-mediated exacerbation of renal injury, apoptosis, and inflammation are attenuated in renal PT NEMO–deficient mice.

**Figure 9:**
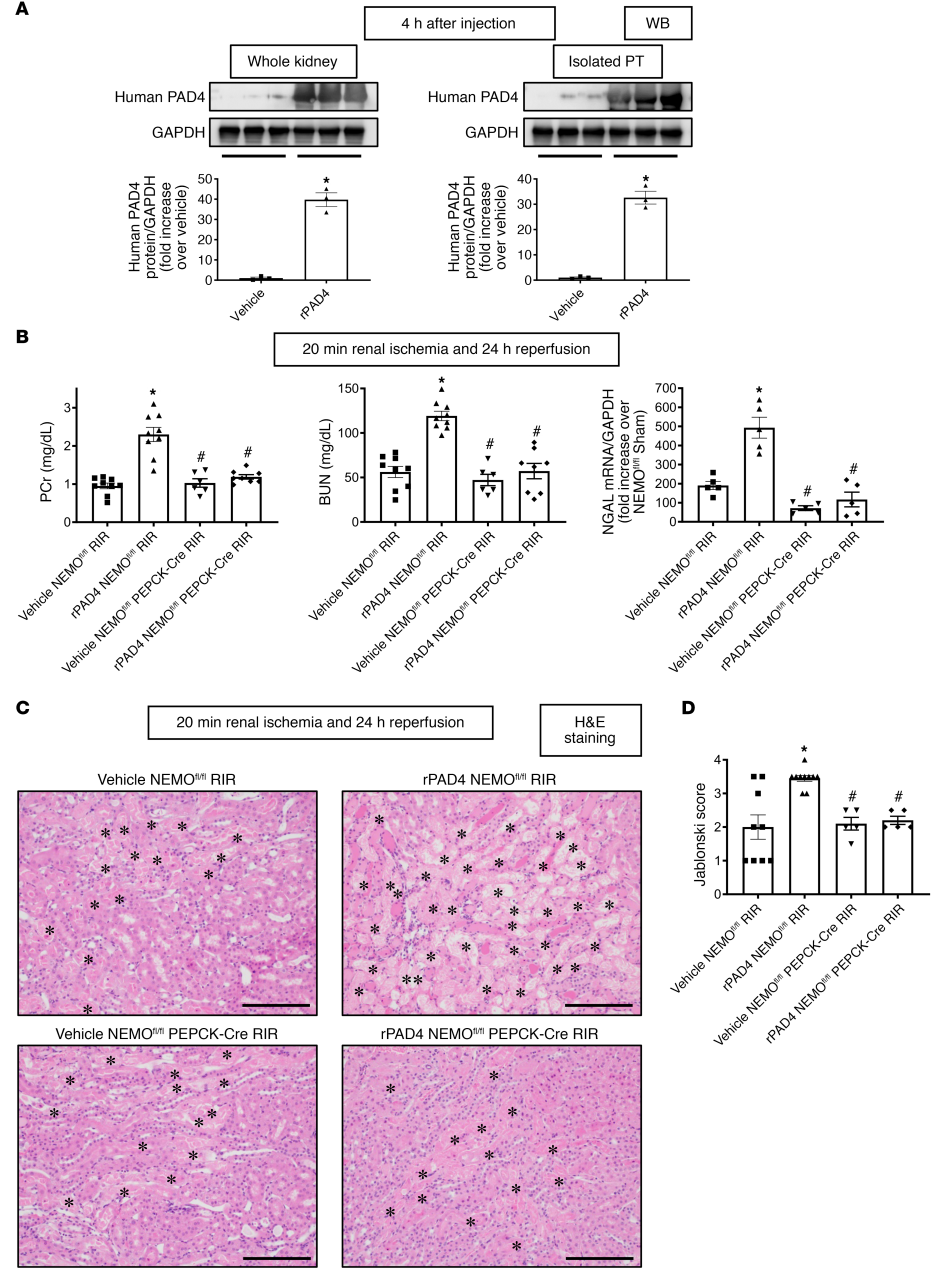
PAD4 exacerbates ischemic AKI in mice via PT NEMO activation. NEMO^fl/fl^ WT or renal PT NEMO–deficient (NEMO^fl/fl^ PEPCK-Cre) mice were treated with vehicle (saline) or with recombinant PAD4 (rPAD4, 10 μg i.v. 15 minutes before renal ischemia) and subjected to sham surgery or to 20 minutes of renal ischemia and 24 hours of reperfusion (RIR, *N* = 5–9). rPAD4 treatment increased human PAD4 protein expressions (**A**) and renal injury measured by plasma creatinine, BUN, and kidney NGAL mRNA (**B**) and renal tubular necrosis (**C** and **D**) in NEMO^fl/fl^ mice. Severe tubular necrosis and proteinaceous casts (*) are indicated in H&E images (×200 original magnification images). Scale bar: 200 μm. For statistical analysis of human PAD4 protein expressions (**A**), Student’s *t* test was used to detect significant changes. For other statistical analysis (**B** and **D**), 1-way ANOVA plus Tukey’s post hoc multiple-comparisons test was used to detect significant changes. Scale bar: 200 μm *P < 0.05 versus vehicle-treated NEMO^fl/fl^ mice subjected to renal IR injury. ^#^*P* < 0.05 versus rPAD4-treated NEMO^fl/fl^ mice subjected to renal IR injury. Error bars represent 1 SEM.

We previously demonstrated that rPAD4 treatment exacerbated ischemic AKI in mice (12, 13). To determine whether human rPAD4 enters kidney and renal proximal tubular cells, mice were treated with 10 μg rPAD4. We determined that rPAD4 treatment increased human PAD4 protein expression in both whole kidney and primary cultures of proximal tubules compared with vehicle-treated mouse kidney and proximal tubules (Figure 9A). Next, to determine whether rPAD4 targets renal PT NEMO to exacerbate ischemic AKI, we treated NEMO^fl/fl^ control mice and renal PT NEMO–null mice with 10 μg human rPAD4 15 minutes before 20 minutes of renal IR injury. rPAD4 treatment exacerbated injury in NEMO^fl/fl^ mice subjected to 20 minutes renal IR (*N* = 5–9, Figure 9B). In contrast, rPAD4 failed to increase renal injury of mice deficient in renal PT NEMO subjected to 20 minutes of renal IR (*N* = 5–8).

**Figure 10:**
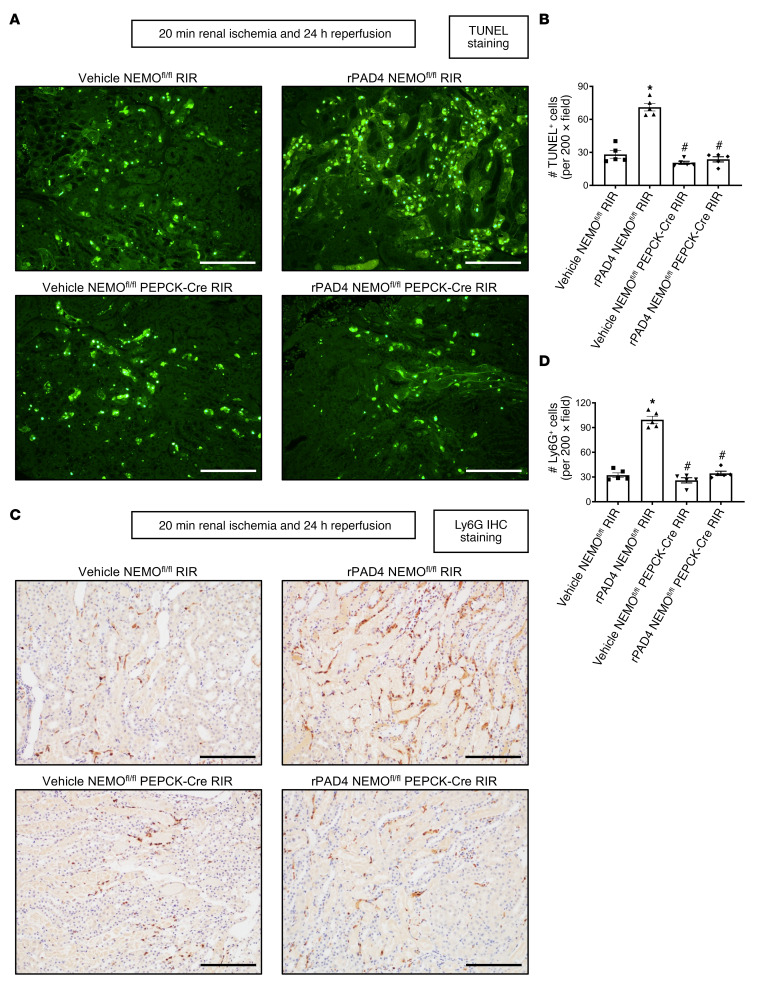
PAD4 exacerbates ischemic AKI-induced renal tubular apoptosis and neutrophil infiltration in mice via PT NEMO activation. NEMO^fl/fl^ WT or renal PT NEMO–deficient (NEMO^fl/fl^ PEPCK-Cre) mice were treated with vehicle (saline) or with recombinant PAD4 (rPAD4, 10 μg i.v. 15 minutes before renal ischemia) and subjected to sham surgery or to 20 minutes of renal ischemia and 24 hours of reperfusion (RIR, *N* = 5–9). rPAD4 treatment exacerbated renal tubular apoptosis (**A** and **B**) and neutrophil infiltration (**C** and **D**) in NEMO^fl/fl^ mice. TUNEL and neutrophil IHC show ×200 original magnification images. Scale bar: 200 μm. For statistical analysis, 1-way ANOVA plus Tukey’s post hoc multiple-comparisons test was used to detect significant changes. Scale bar: 200 μm *P < 0.05 versus vehicle-treated NEMO^fl/fl^ mice subjected to renal IR injury. ^#^*P* < 0.05 versus rPAD4-treated NEMO^fl/fl^ mice subjected to renal IR injury. Error bars represent 1 SEM.

**Figure 11:**
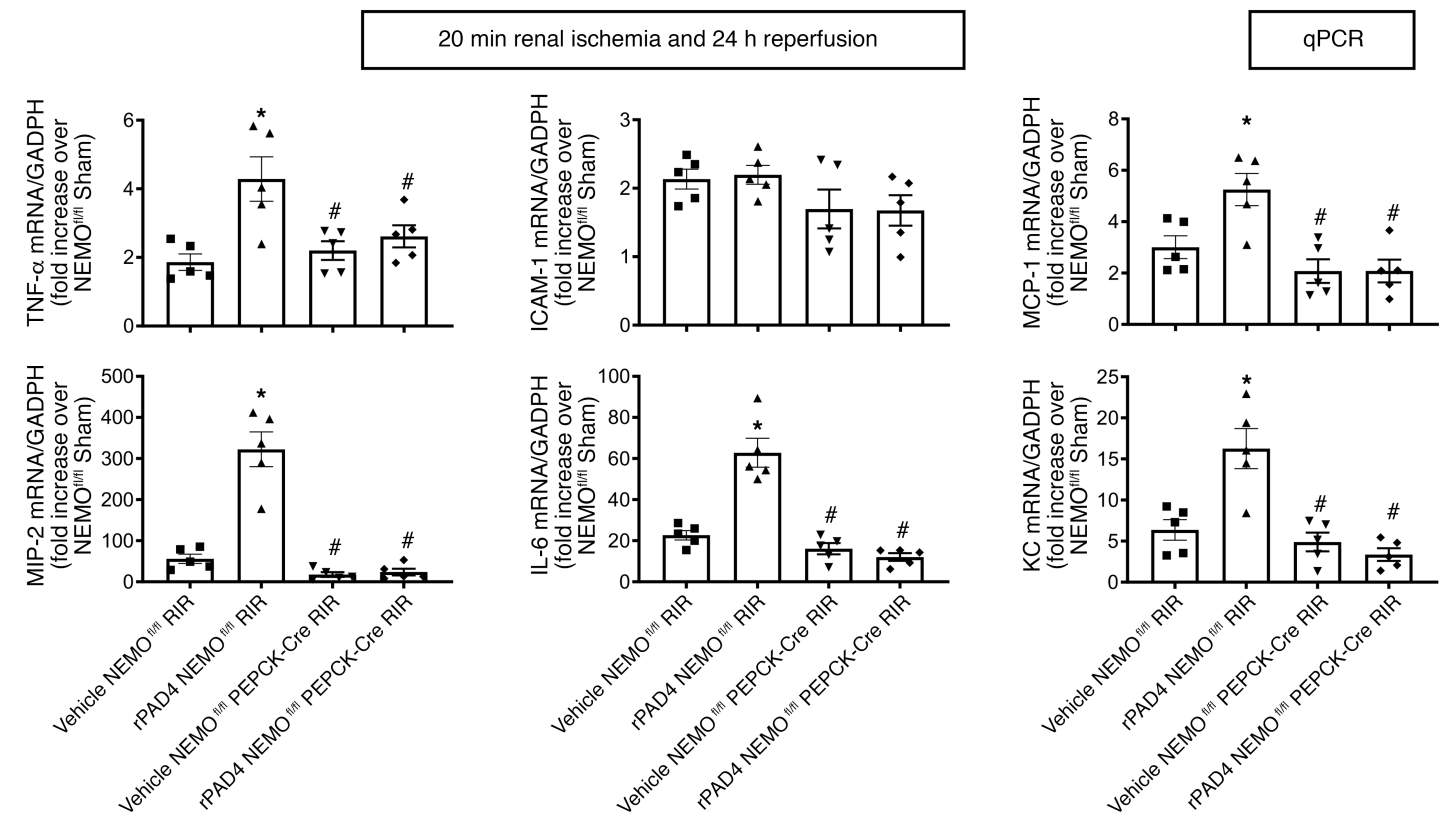
PAD4 exacerbates ischemic AKI-induced renal tubular inflammatory cytokine generation in mice via PT NEMO activation. NEMO^fl/fl^ WT or renal PT NEMO–deficient (NEMO^fl/fl^ PEPCK-Cre) mice were treated with vehicle (saline) or with recombinant PAD4 (rPAD4, 10 μg i.v. 15 minutes before renal ischemia) and subjected to sham surgery or to 20 minutes of renal ischemia and 24 hours of reperfusion (RIR, *N* = 5–9). rPAD4 treatment exacerbated kidney proinflammatory cytokines and chemokines except ICAM-1 mRNA induction in NEMO^fl/fl^ mice. For RT-PCR, fold increases in proinflammatory mRNAs normalized to GAPDH from quantitative RT-PCR reactions for each indicated mRNA are shown. For statistical analysis, 1-way ANOVA plus Tukey’s post hoc multiple-comparisons test was used to detect significant changes. Scale bar: 200 μm *P < 0.05 versus vehicle-treated NEMO^fl/fl^ mice subjected to renal IR injury. ^#^*P* < 0.05 versus rPAD4-treated NEMO^fl/fl^ mice subjected to renal IR injury. Error bars represent 1 SEM.

As expected, the kidneys of NEMO^fl/fl^ control mice treated with 10 μg i.v. rPAD4 and subjected to renal IR showed increased tubular necrosis (*N* = 5–9, Figure 9, C and D), renal tubular apoptosis (Figure 10, A and B), neutrophil infiltration (Figure 10, C and D), and kidney cytokine mRNA induction (Figure 11). In contrast, rPAD4 treatment did not increase tubular necrosis (*N* = 5, Figure 9, C and D), renal tubular apoptosis (Figure 10, A and B), neutrophil infiltration (Figure 10, C and D), or kidney cytokine mRNA induction (Figure 11) in renal PT NEMO–null mice.

### Induction of canonical NF-κB–mediated proinflammatory signaling with NEMO activation.

**Figure 12:**
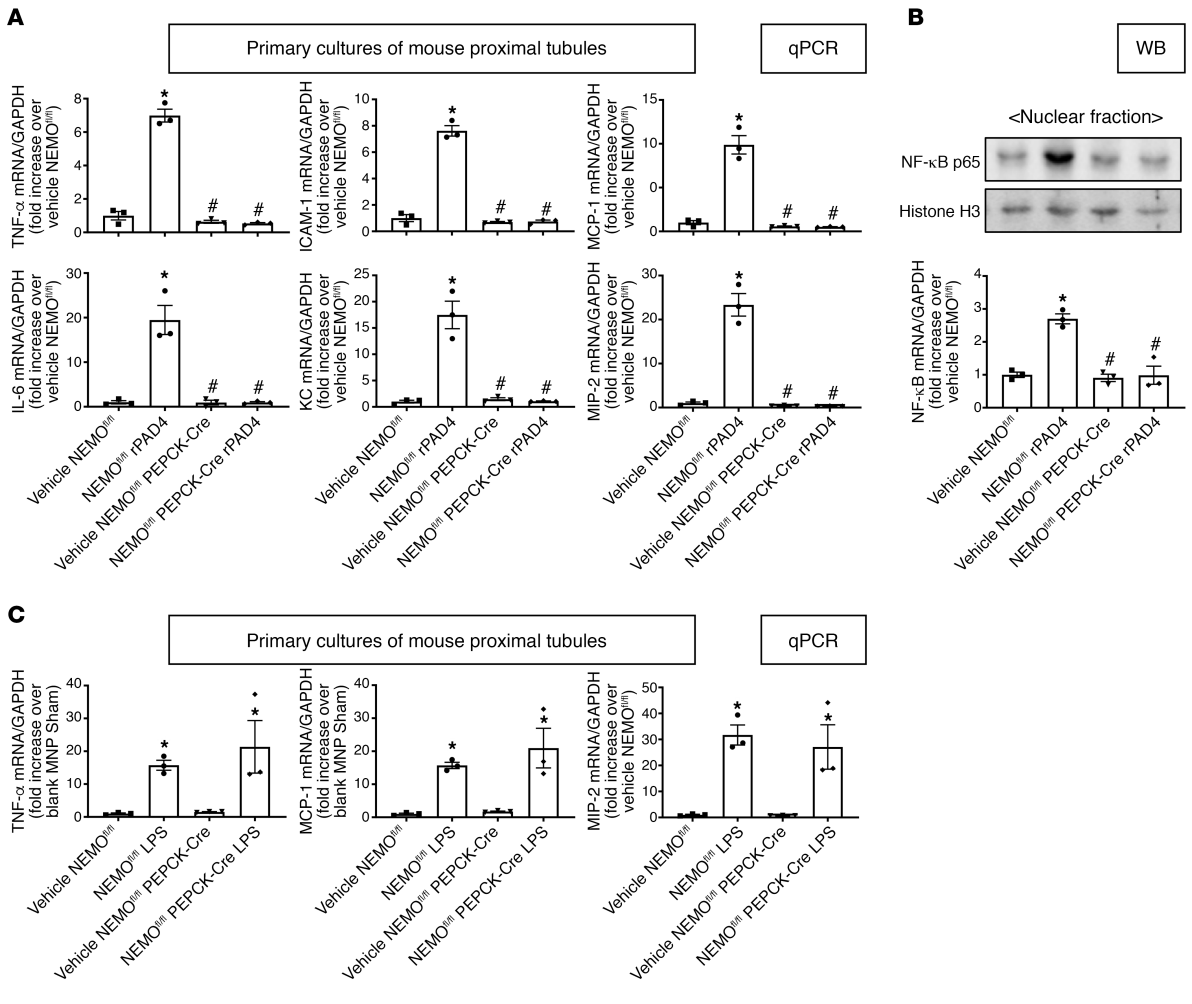
NEMO-mediated induction of renal proximal tubular NF-κB proinflammatory signaling in mouse renal proximal tubule cells. Primary cultures of renal proximal tubule cells from NEMO^fl/fl^ WT or renal PT NEMO–deficient (NEMO^fl/fl^ PEPCK-Cre) mice were treated with recombinant PAD4 (rPAD4, 10 μg/mL) for 6 hours (**A**) or 4 hours (**B**) or with lipopolysaccharides (LPS, 10 μg/mL) for 6 hours (**C**). (**A**) With RT-PCR, we measured the expressions of TNF-α, ICAM-1, MCP-1, IL-6, KC, and MIP-2 mRNAs. Fold increases in mRNAs normalized to GAPDH from quantitative RT-PCR reactions for each indicated mRNA (*N* = 3) are shown. (**B**) A representative immunoblotting experiment for nuclear p65 NF-κB subunit (top) and band intensity quantifications normalized to histone H3 (*N* = 3, bottom). (**C**) With RT-PCR, we measured the expression of TNF-α, MCP-1, and MIP-2 mRNAs. Fold increases in mRNAs normalized to GAPDH from quantitative RT-PCR reactions for each indicated mRNA (*N* = 3) are shown. For statistical analysis, 1-way ANOVA plus Tukey’s post hoc multiple-comparisons test was used to detect significant changes. *P < 0.05 versus vehicle-treated NEMO^fl/fl^ mice. ^#^*P* < 0.05 versus rPAD4-treated NEMO^fl/fl^ mice. Error bars represent 1 SEM.

To determine whether PAD4 activates renal proximal tubular proinflammatory signaling via NEMO activation, we treated renal proximal tubule cells isolated from NEMO^fl/fl^ mice or renal PT NEMO–null mice with rPAD4 (10 μg/mL) for 6 hours. Figure 12A shows significant inductions of TNF-α, ICAM-1, MCP-1, IL-6, KC, and MIP-2 mRNA in primary cultures of NEMO^fl/fl^ renal proximal tubule cells treated with rPAD4 (*N* = 3). In contrast, rPAD4 failed to induce these proinflammatory cytokine mRNAs in renal proximal tubules cells from renal PT NEMO–null mice. In addition, rPAD4 induced NF-κB p65 subunit nuclear translocation in renal proximal tubule cells from NEMO^fl/fl^ mice but not from renal PT NEMO–null mice (Figure 12B, N** = 3).

We also treated primary cultures of renal proximal tubule cells with TNF-α or with LPS that can activate both canonical (classical) and noncanonical (transcription factor RelB or NEMO independent) NF-κB pathways (18–20). Figure 12C shows that LPS induced TNF-α, MCP-1, and MIP-2 mRNA expression in primary renal proximal cell cultures from NEMO^fl/fl^ and renal PT NEMO–null mice. Similarly, NEMO deficiency did not attenuate TNF-α–mediated proinflammatory cytokine induction in mouse renal tubule cells (data not shown).

**Figure 13:**
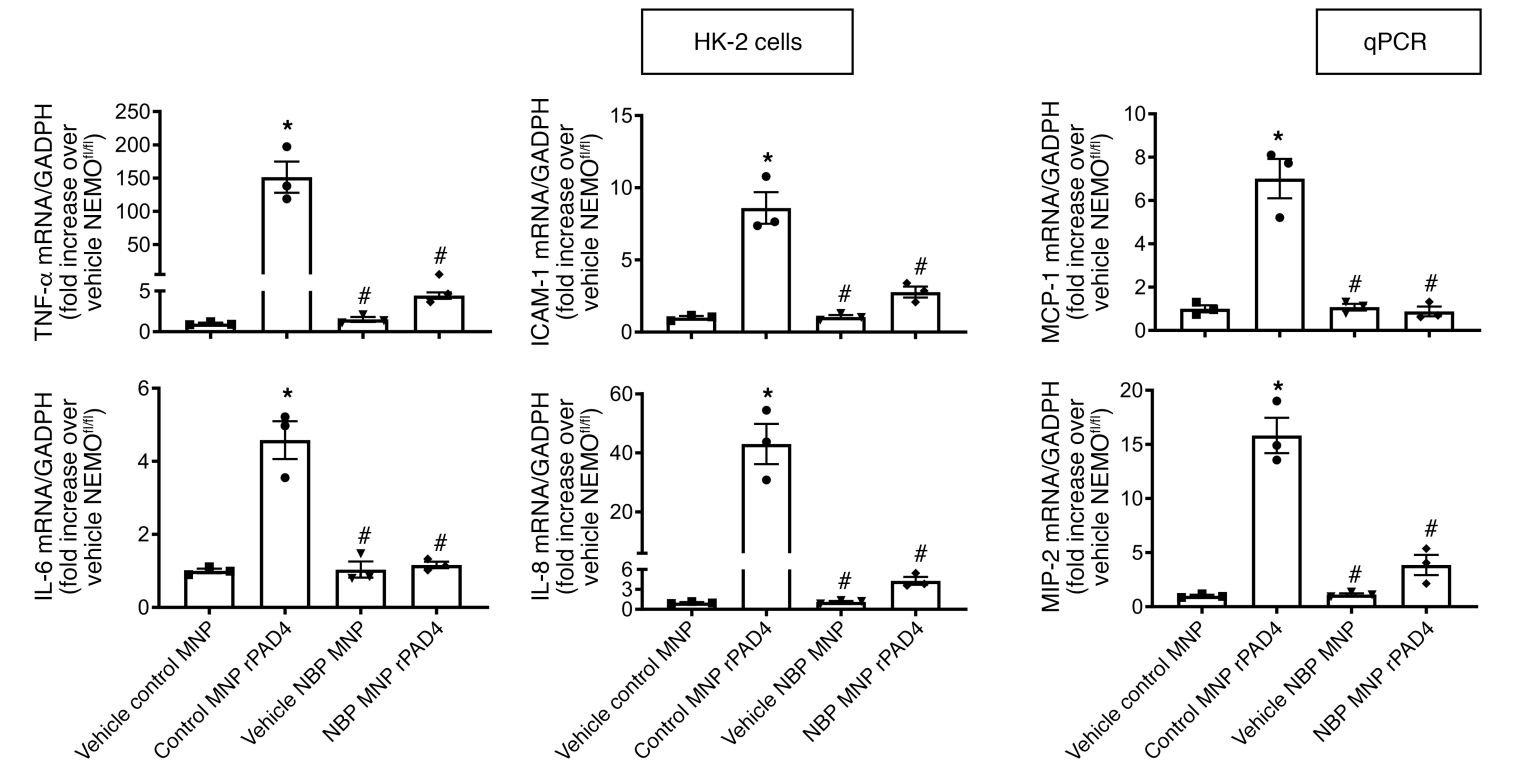
NEMO-mediated induction of renal proximal tubular NF-κB proinflammatory signaling in human renal proximal tubule cells. Human proximal tubule (HK-2) cells were treated with 10 μg/mL rPAD4 for 6 hours with or without pretreatment of 10 μM NBP MNPs (*N* = 3). The expressions of TNF-α, ICAM-1, MCP-1, IL-6, IL-8, and MIP-2 mRNAs were measured with RT-PCR. Fold increases in mRNAs normalized to GAPDH from quantitative RT-PCR reactions for each indicated mRNA (*N* = 3) are shown. For statistical analysis, 1-way ANOVA plus Tukey’s post hoc multiple-comparisons test was used to detect significant changes. *P < 0.05 versus control MNP-treated cells. ^#^*P* < 0.05 versus control MNP-treated cells. Error bars represent 1 SEM.

To complement the gene deletion studies and show specific inhibition of the NEMO signaling pathway in renal tubular cells treated with NBP MNP, human proximal tubule (HK-2) cells were treated with rPAD4 for 6 hours with or without pretreatment of 10 μM NBP MNP (*N* = 3). We showed that NBP MNP completely prevented rPAD4-mediated induction of TNF-α, ICAM-1, MCP-1, IL-6, IL-8, and MIP-2 mRNA (Figure 13).

**Figure 14:**
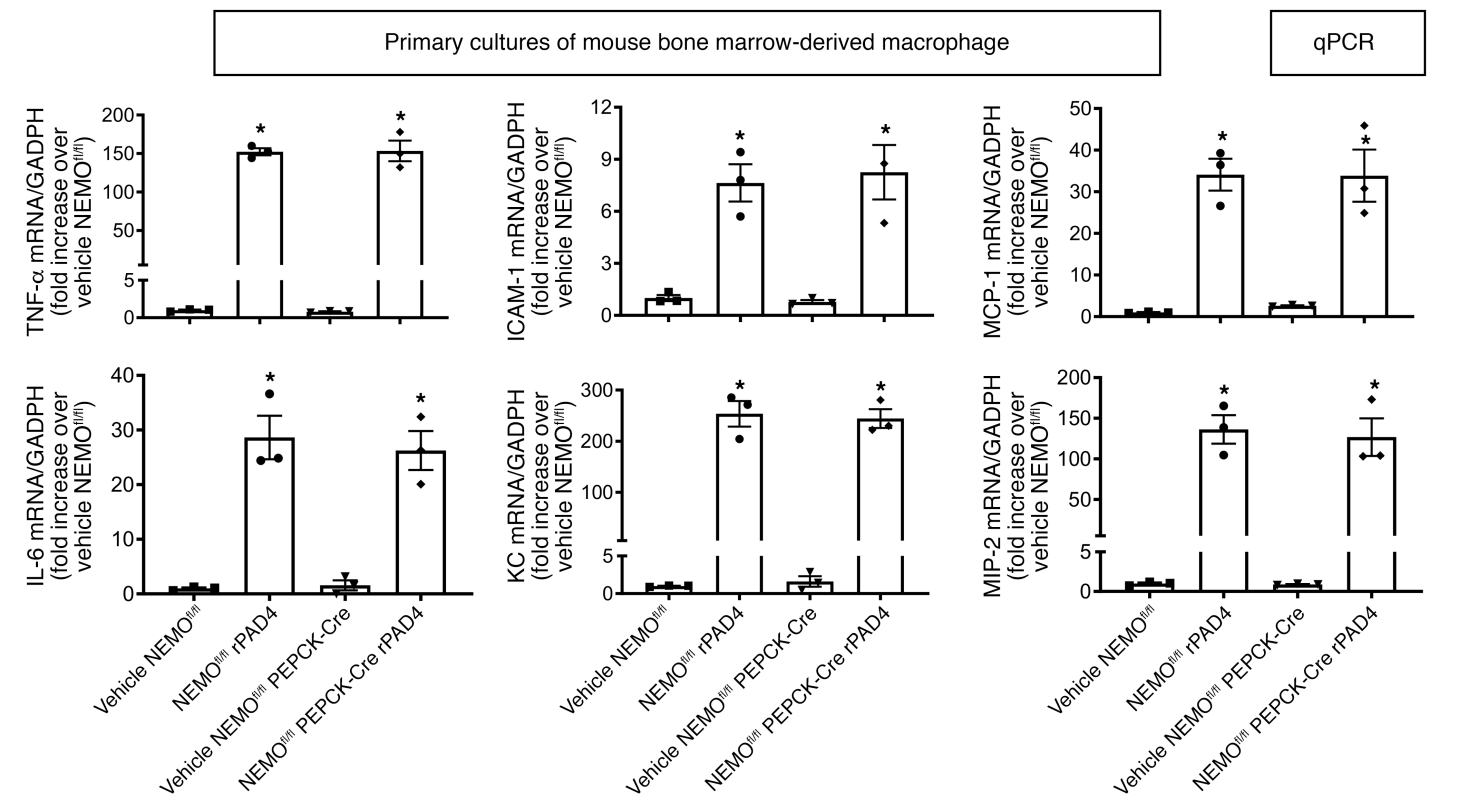
Renal PT NEMO does not mediate bone marrow–derived macrophage proinflammatory signaling. Freshly isolated bone marrow–derived macrophages from NEMO^fl/fl^ WT or renal PT NEMO–deficient (NEMO^fl/fl^ PEPCK-Cre) mice were treated with recombinant PAD4 (rPAD4, 10 μg/mL) for 6 hours and we measured the expression of TNF-α, ICAM-1, MCP-1, IL-6, KC, and MIP-2 mRNAs with RT-PCR. Fold increases in mRNAs normalized to GAPDH from quantitative RT-PCR reactions for each indicated mRNA (*N* = 3) are shown. For statistical analysis, 1-way ANOVA plus Tukey’s post hoc multiple-comparisons test was used to detect significant changes. *P < 0.05 versus vehicle-treated NEMO^fl/fl^ or control MNP-treated cells. Error bars represent 1 SEM.

Finally, we treated primary cultures of bone marrow–derived macrophages isolated from NEMO^fl/fl^ and renal PT NEMO–null mice with 10 μg/mL rPAD4. Demonstrating renal proximal tubule–specific NEMO deletion further, rPAD4 induced proinflammatory cytokine mRNAs in NEMO^fl/fl^ and renal PT NEMO–null mouse bone marrow–derived macrophages (Figure 14).

## Discussion

We previously demonstrated that renal proximal tubule cells not only express nuclear PAD4, but its expression is upregulated after renal IR via ATP-mediated activation of P2X7 receptors (12, 14). Inhibition of genetic deletion of PAD4 attenuated renal IR injury in mice, significantly reducing renal tubular inflammation, necrosis, and apoptosis (12–14). Activated renal tubular PAD4 gets translocated to the cytosol after IR and in the cytosol, PAD4 selectively promotes the activity of one of the key regulators of NF-κB activation IKKγ (also called NEMO) via citrullination, resulting in nuclear translocation of NF-κB (15). NEMO neutralization with a specific binding peptide attenuated PAD4-mediated NF-κB nuclear translocation and inflammatory cytokine generation.

Our previous findings suggest a potential therapeutic role for NBP therapy to protect against ischemic AKI. However, systemic administration of NBP will inhibit NEMO in every cell type in the body and may not be a viable option as a treatment for ischemic AKI. Although kidney NEMO may exacerbate renal IR injury by inducing apoptosis and inflammation, other cell types in the kidney may benefit from NEMO signaling. Indeed, long-term systemic NEMO inhibition or deletion may not produce antiinflammatory effects in all cell types and may produce detrimental effects in extrarenal cell types and organs. For example, lymphocyte NEMO deletion increases intrarenal Th17 cells and exacerbates ischemic AKI in mice (21). In addition, myeloid NEMO deletion results in osteoporosis via upregulation of transcriptional repressors (22). Moreover, NEMO activity is critical for thyroid function, and thyroid-specific NEMO deletion causes hypothyroidism in mice (23). Finally, hepatocyte NEMO deletion causes steatohepatitis and liver cancer in mice (24). These studies show that systemic administration of a NEMO antagonist may have limited therapeutic potential for ischemic AKI.

Since NEMO expression is ubiquitous among many organs and cell types even in the kidney, it remains unclear whether renal PT NEMO plays a critical role in ischemic AKI and subsequent renal tubular inflammation. Our current study showed that renal PT NEMO (IKKγ) plays a critical role in ischemic AKI by promoting renal tubular inflammation, resulting in increased renal tubular cell death, apoptosis, and neutrophil infiltration. Our findings are based on 2 distinct approaches: 1) we created mice with renal tubular–specific deletion of NEMO and 2) we selectively delivered NBP to renal proximal tubule cells using NBP encapsulated in MNPs. Mice deficient in renal PT NEMO and mice treated with NBP MNP were significantly protected against ischemic AKI, having reduced renal tubular inflammation, necrosis, and apoptosis.

Renal tubular NF-κB activation leads to transcription of several proinflammatory cytokines, including neutrophil-attracting IL-8/KC and MIP-2 (also called CXCL8 and CXCL2, respectively) (25, 26). IKKγ, also known as NEMO, is a key regulator of NF-κB (27, 28). Indeed, NF-κB activation plays a critical role in generating the inflammatory response as a key transcription factor for several proinflammatory chemokines. However, NF-κB is also a critical transcription factor for cell survival, proliferation, and differentiation (29, 30). Therefore, global NF-κB inhibition may decrease the renal inflammatory response after IR, but also may reduce renal cell proliferation and survival (31). Therefore, selective inhibition of the proinflammatory NF-κB signaling pathway would be required to protect against the inflammatory response after renal IR. Relevant to potential therapy, selective NEMO inhibition with NBP selectively attenuates proinflammatory gene expression and improves kidney injury without negative effects on renal tubular cell proliferation and survival (15). Interestingly, Markó et al. demonstrated that selective IκBα suppression leading to NF-κB inhibition in tubular epithelial cells protected against ischemic kidney injury with reduced apoptotic/necrotic tubular cell death and inflammation (32). Their study agrees with the renal protective effects of IKKγ deletion or inhibition in mice subjected to ischemic AKI in our study. We showed here that renal PT NEMO deletion resulted in total loss of rPAD4-mediated proinflammatory cytokine mRNA induction in renal proximal tubule cells. In contrast, renal PT NEMO deletion failed to attenuate proinflammatory cytokine mRNA induction in response to TNF-α or to LPS, further demonstrating that PAD4 selectively activated the canonical NF-κB pathway via NEMO citrullination. Furthermore, NBP MNP blocked rPAD4-mediated proinflammatory cytokine induction in human renal proximal tubule cells. Taken together, our findings further support that PAD4 induces inflammatory cytokine signaling in renal proximal tubular cells via NEMO-dependent mechanisms and that renal PT NEMO plays a critical role in proinflammatory cytokine regulation via the canonical NF-κB pathway.

Many promising preclinical studies failed to show efficacy in the clinical setting (33, 34). A major factor in the lack of successful translation from bench research to clinical therapy is clearly the complex nature of clinical AKI in patients with multiple comorbidities in comparison with simplified laboratory models of AKI in healthy animals (35, 36). Another reason for lack of success may be that systemic administration of drugs to treat clinical AKI will target every organ system and multiple cell types. Drugs that protect renal epithelial cells may produce unwanted detrimental effects in other cell types (e.g., myeloid cells, hepatocytes) as discussed above. Finally, drugs given at nontoxic doses may not achieve therapeutically adequate levels to treat or prevent ischemic AKI. Here, we used a strategy to selectively deliver NBP in adequate therapeutic levels in renal proximal tubular cells with NBP MNP. We previously showed that systemic injection of 5 mg/kg NBP in mice subjected to renal IR protected against ischemic AKI (15). It is exciting and clinically relevant that MNP encapsulating a significantly lower dose of NBP (8 or 16 μg/kg NBP) compared with doses used for systemic injection provided powerful protection against ischemic AKI in mice by attenuating renal tubular necrosis, inflammation, and apoptosis. Furthermore, we determined that NBP MNP delivered 15 minutes after reperfusion was almost as protective as NBP MNP given 6 hours before renal ischemia, providing a basis for even greater clinical significance.

MNPs are PEG-coated poly(lactic-co-glycolic acid) (PLGA) polymer particles with diameters of approximately 300–400 nm (37, 38). We showed previously that MNPs localize to the basolateral region of proximal tubule epithelial cells preferentially over other organs (> 30-fold selectivity) and remain in the kidney for approximately 7 days after injection (37, 39). These findings have significant clinical potential in that renal proximal tubular cells are the major site of injury in ischemic AKI (40, 41). Also clinically relevant, MNP treatment did not have a detrimental effect on renal or hepatic function, inflammation, or hematological problems (37, 38, 42). Moreover, we recently showed with intravital imaging studies that kidneys subjected to renal IR maintained approximately 30-fold kidney-specific delivery of MNPs compared with other organ studies (39). Therefore, it appears that renal tubular necrosis and inflammation have no impact on kidney-selective delivery of MNPs.

One potential concern may be that PEPCK-Cre recombinase is expressed in renal proximal cells and in a subset of periportal hepatocytes (16). Indeed, we showed in this study that hepatic NEMO mRNA expression was approximately 32% less in NEMO^fl/fl^ PEPCK-Cre mice compared with NEMO^fl/fl^ mice. In contrast, renal proximal tubules from NEMO^fl/fl^ PEPCK-Cre mice had a greater than 99% reduction in NEMO mRNA and a greater than 96% reduction in NEMO protein expression compared with NEMO^fl/fl^ mice. Therefore, NEMO^fl/fl^ PEPCK-Cre mice showed near-complete deletion of renal PT NEMO and partial reduction in liver NEMO. This is consistent with how PEPCK-Cre mice were generated. The PEPCK-Cre transgene was generated using a mutated version of the PEPCK promoter, which reduces PEPCK expression in the liver by 60% and increases PEPCK expression in the kidney by 10-fold in transgenic mice (17). We do not believe that partial reduction in hepatic NEMO contributed to attenuated ischemic AKI observed in NEMO^fl/fl^ PEPCK-Cre mice because ablation of hepatic NEMO potentiates hepatotoxicity and spontaneously induces liver inflammation, steatosis, and fibrosis (24, 43). Further supporting this, we demonstrated that WT and PT NEMO–deficient mice developed a similar degree of liver injury after renal IR. Therefore, we conclude that renal PT NEMO deletion or inhibition protects against ischemic AKI independent of hepatic NEMO.

Our previous and current study demonstrated kidney-selective delivery of NBP MNP of 27–94-fold more than other organs, including the spleen (37). Moreover, we showed renal proximal tubular delivery of MNP with PEG/PHA lectin costaining (Figure 1C). PEG staining did not occur in extrarenal organs. Therefore, it is most likely that the reduction in inflammation and attenuation of kidney-infiltrating proinflammatory cells was due to reduced ischemic AKI and decreased proinflammatory response. However, we cannot completely rule out the impact of NBP MNP (no matter how minor) on extrarenal inflammatory cells.

**Figure 15:**
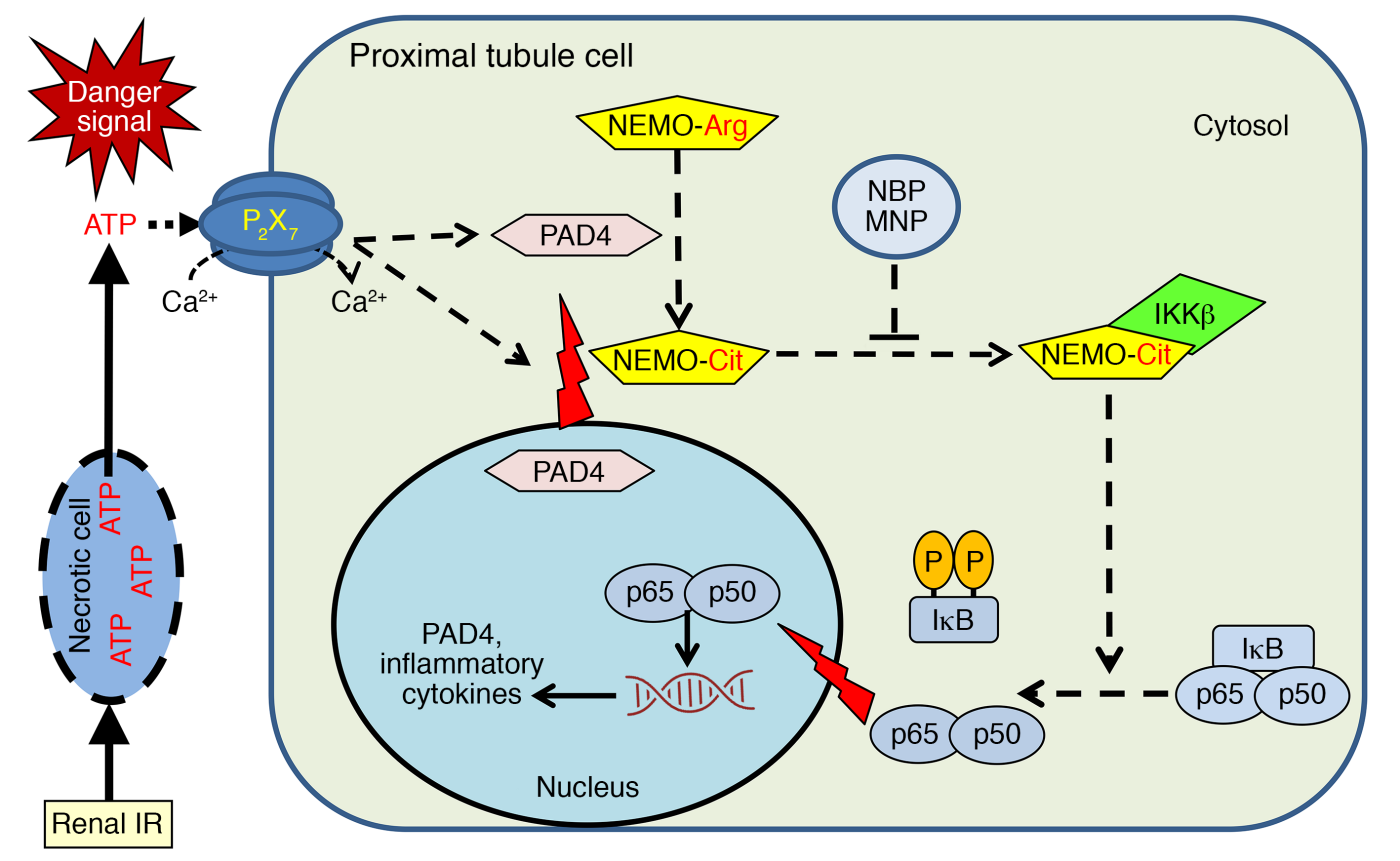
Overview of NEMO-mediated renal tubular inflammation and injury. ATP released by necrotic or dying renal cells activates P2X7 purinergic receptors to induce renal tubular PAD4. Cytosolic translocation of PAD4 after renal IR preferentially citrullinates NEMO, which in turn drives proinflammatory NF-κB signaling with increased cytokine/chemokine synthesis and neutrophil infiltration. NEMO inhibition with MNP-encapsulated NBP protects against ischemic AKI and decreases renal tubular proinflammatory cytokine induction.

In summary, we demonstrated in this study that renal PT NEMO plays a critical role in ischemic AKI by promoting kidney tubular necrosis, inflammation, and apoptosis. We also demonstrated in this study a potentially novel and innovative method to treat ischemic AKI using NBP MNP. Our study suggests that kidney-targeted MNP-mediated selective drug delivery is an exciting method to treat AKI with improved therapeutic specificity and potentially reduced systemic toxicity by allowing a lower drug dosage. Our previous and current findings allow us to propose a summary of PAD4-mediated renal tubular inflammation and exacerbation of ischemic AKI (Figure 15). We showed that ATP released by necrotic or dying renal cells activated P2X7 purinergic receptors to induce renal tubular PAD4. Cytosolic translocation of PAD4 after renal IR preferentially citrullinated NEMO, which in turn drove proinflammatory NF-κB signaling with increased cytokine/chemokine synthesis and neutrophil infiltration. NEMO inhibition with NBP MNP protected against ischemic AKI and decreased renal tubular proinflammatory cytokine induction.

## Methods

### Generation of mice with renal proximal tubule cell–specific NEMO deficiency.

We bred mice with loxP-flanked Nemo alleles (NEMO^fl/fl^ mice; ref. 44) with mice that expressed Cre recombinase selectively in proximal tubular epithelia (Cre recombinase under the control of PEPCK-Cre — generated by Volker Haase, Vanderbilt University, Nashville, Tennessee, USA; ref. 17). All mice were backcrossed with C57BL/6 mice for at least 6 generations. This approach allowed us to generate sibling mice with proximal tubule–specific deletion of NEMO (NEMO^fl/fl^ PEPCK-Cre mice) or WT (NEMO^fl/fl^) mice. Tail PCR with PEPCK-Cre and NEMO loxP-specific primers (Table 1) confirmed the genotypes of proximal tubule cell–specific NEMO-null mice and the control WT mice (NEMO^fl/fl^) generated from breeding.

### Confirmation of renal PT NEMO deletion in NEMO^fl/fl^ PEPCK-Cre mice.

We confirmed selective deletion of renal PT NEMO in NEMO^fl/fl^ PEPCK-Cre mice by measuring NEMO mRNA with RT-PCR and NEMO protein expression with immunoblotting using anti-NEMO antibody (ab178872, Abcam) in isolated proximal tubule cells (45) as described previously (46, 47). We also measured NEMO mRNA in bone marrow–derived macrophages and the kidney, spleen, liver, and small intestine. Primer design was based on published GenBank sequences (Table 1). To control for RNA loading, GAPDH mRNA expression was also measured.

### Generation of MNPs and incorporation of NBP.

MNPs encapsulating NBP (MilliporeSigma, 480025) were formulated similarly to previously described methods with minor modifications (37, 38). Briefly, 38–54 kDa molecular weight PLGA (MilliporeSigma) was conjugated to 5 kDa carboxylic acid–terminated PEG (Nanocs) before particle formulation. The conjugated copolymer (100 mg) was dissolved in 2 mL acetonitrile. Then, 100 μg of NBP in water was added to the copolymer solution and bath sonicated for 2 minutes. The resultant emulsion was added dropwise to a solution of purified water (4 mL) and Pluronic F-68 (75 μL; Thermo Fisher Scientific). After 2 hours of stirring, the particle solution was centrifuged at 5400*g* for 15 minutes. The nanoparticle pellet was washed with 10 mL purified water and centrifuged under the same specifications. The resultant pellet was resuspended in a 2% sucrose solution and lyophilized for storage at –20°C. Empty control MNPs were formulated identically to NBP MNP as above without emulsion with the peptide.

The hydrodynamic diameter and PDI of the MNPs was characterized via dynamic light scattering (Malvern) in a 10 mg/mL PBS suspension. To quantify peptide loading into the particles, approximately 10 mg lyophilized particle powder was dissolved in 200 μL acetonitrile and shaken at room temperature for 30 minutes. To this solution, we added 300 μL Tris-EDTA buffer (Thermo Fisher Scientific) before centrifugation at 31,000*g* for 30 minutes. The supernatant containing liberated peptide was used for quantification via the MicroBCA Assay Kit (Thermo Fisher Scientific) according to the manufacturer’s instructions.

### PEG IHC to test renal proximal tubular delivery of MNPs.

Because the surface of MNPs is composed of PEG, we performed florescent IHC for PEG to detect renal proximal tubular localization of MNPs administered. Kidneys from mice treated with 38 mg/kg NBP MNP 6 hours before renal IR injury were fixed with 4% paraformaldehyde, dehydrated with 30% sucrose, frozen in OCT (Tissue-Tek), and cryosectioned (5 μm). Kidney sections were permeabilized with 0.2% Triton X-100 for 10 minutes and the sections were then autoclaved in 10 mM sodium citrate, pH 6.0, for 10 minutes to retrieve antigens. After blocking sections with PBS containing 10% normal rabbit serum, sections were incubated with 1:100 anti-PEG antibody (ab94764, Abcam) specific to the PEG backbone plus PHA lectin antibody (proximal tubule–specific marker, Molecular Probes) or plus aquaporin-2 antibody (collecting duct–specific marker, AQP-002, Alomone Labs). After incubating sections with specific fluorescent secondary antibodies (Thermo Fisher Scientific), kidney slides were counterstained with DAPI to visualize cell nuclei, mounted with Vectashield (Vector) mounting media, and imaged with a fluorescent microscope (Olympus IX81). We also performed PEG florescent IHC in the lung, spleen, and liver.

### Renal IR injury in mice.

After Columbia University IACUC approval, 8–10-week-old male PT NEMO–deficient (NEMO^fl/fl^ PEPCK-Cre) mice, control WT (NEMO^fl/fl^) mice, or C57BL/6 mice (The Jackson Laboratory) weighing 20–25 g were anesthetized with i.p. pentobarbital (MilliporeSigma: 50 mg/kg body weight or to effect). Mice were then subjected to right nephrectomy and 30 minutes of left renal ischemia as described previously (48, 49). C57BL/6 mice received i.v. NBP MNPs (19 or 38 mg/kg) or empty MNPs 6 hours before renal ischemia or 15 minutes after reperfusion based on previous studies (37, 39). Previous studies showed that the dosing ranges of approximately 25–50 mg/kg MNP had the greatest accumulation/kidney selectivity profile for the nanoparticles (~27–94-fold kidney selectivity over other organs) (37). We previously showed that exogenous PAD4 exacerbates renal IR injury (12). To test whether exogenous PAD4 targets renal PT NEMO to exacerbate ischemic AKI, PT NEMO–deficient or control (NEMO^fl/fl^) mice were pretreated with human rPAD4 (10 μg, i.v., Cayman Chemical) 15 minutes before 20 minutes of renal IR injury. To determine whether recombinant rPAD4 enters kidney and renal proximal tubular cells, human PAD4 protein expressions in both whole kidney and primary cultures of proximal tubules of mice were measured by Western blotting using anti–human PAD4 antibody (ab128086, Abcam) 4 hours after rPAD4 treatment. Sham-operated animals underwent anesthesia followed by laparotomy, right nephrectomy, bowel manipulations, and wound closure without renal ischemia. Body temperatures of all mice were sustained at approximately 37°C using a surgical heating pad during surgery and during recovery from anesthesia. For pain management, all mice received 0.1–1 mg/kg s.c. sustained-release buprenorphine before surgery. Plasma and kidneys were collected 24 hours after renal IR injury to examine renal dysfunction (plasma creatinine, BUN, and histology); inflammation (neutrophil infiltration, cytokine mRNAs); and apoptosis (TUNEL staining).

### Detection of renal injury and hepatic injury after renal IR.

Twenty-four hours after renal IR injury or sham surgery, we measured plasma BUN, creatinine, and ALT using an enzymatic reagent kit (Thermo Fisher Scientific). This method of plasma creatinine assay limits the interferences from mouse plasma chromogens known to occur in the Jaffe method. We also performed qRT-PCR for kidney NGAL mRNA from mice subjected to sham surgery or to renal IR injury. NGAL is an early and sensitive marker of renal tubular injury (50).

### Histological detection of kidney and liver injury.

Twenty-four hours after renal IR injury or sham surgery, kidney H&E sections were assessed using a grading scale of kidney necrotic IR injury to the proximal tubules (0–4, Renal Injury Score) as outlined by Jablonski et al. (51). The renal pathologist was blinded to the experimental conditions. Deidentified slides were H&E-stained coronal cross sections of bivalved whole kidney showing full-thickness cortex and medulla. The cortical and medullary parenchyma was evaluated in its entirety in all the microscopic fields covering the entire slide to generate the Jablonski score. We also examined liver H&E sections for differences in hepatic histological injury between WT mice and renal PT NEMO–deficient mice.

### Detection of kidney apoptosis.

Twenty-four hours after sham surgery or renal IR injury, TUNEL staining detected fragmented DNA as described (52) using a commercially available kit (Roche). Apoptotic TUNEL-positive cells were quantified in 5–7 randomly chosen ×200 original magnification microscope image fields in the corticomedullary junction, and results were expressed as apoptotic cells counted per field.

### Detection of kidney neutrophil infiltration.

Kidney neutrophil infiltration after IR injury or sham surgery was detected with IHC staining using rat anti–mouse Ly6G monoclonal antibody (14-5931-85, Thermo Fisher Scientific) as described (46, 53). Primary IgG_2a_ antibody (MCA1212, AbD Serotec) was used as a negative isotype control. Quantification of kidney-infiltrating neutrophils was performed using 5–7 randomly chosen ×200 original magnification microscope image fields (corticomedullary junction for kidney neutrophils), and results were expressed as neutrophils counted per field.

### IHC staining for NF-κB and p-NF-κB.

We detected NF-κB and p-NF-κB expression after IR injury or sham surgery with IHC staining using rabbit anti–NF-κB and anti–p-NF-κB antibodies (8242S, 3033S, Abcam) as described (46, 53). NF-κB–positive tubular cells in nuclear and p-NF-κB expression were quantified from 3 to 5 randomly chosen ×200 original magnification microscope image fields as described by Ruifrok et al. (54).

### qRT-PCR for proinflammatory cytokine and chemokine mRNA expression.

Renal inflammation after IR was also assessed by measuring proinflammatory mRNA markers, including IL-6, ICAM-1, MCP-1, KC, MIP-2, and TNF-α qRT-PCR, as described previously with primers listed in Table 2 (46). Primer design was based on published GenBank sequences. To confirm equal RNA input, GAPDH mRNA and relative expression of proinflammatory mRNA were calculated with the ΔΔCt method. qRT-PCR was performed using MyiQ Real Time Detection System (Bio-Rad) using FastStart Universal SYBR Green Master (ROX) (Roche).

### ELISA for MCP-1 and MIP-2.

Plasma MCP-1 and MIP-2 levels were measured with mouse-specific ELISA kits 24 hours after sham or renal IR surgery (Thermo Fisher Scientific).

### Cell culture and in vitro experiments.

Mouse kidney proximal tubules from control (NEMO^fl/fl^) or PT NEMO–null mice were isolated using Percoll density gradient separation and grown as described previously (45). Bone marrow–derived macrophages were isolated from NEMO^fl/fl^ or PT NEMO–null mice as previously described (55). Mouse hind limb femurs and tibiae were dissected, and marrow plugs were dispersed by passing a 26-gauge needle through them, and the cells were suspended by vigorous pipetting and washed. Cells were cultured in DMEM/F12 medium supplemented with 100 IU/mL penicillin, 100 μg/mL streptomycin, 10% FBS, and 10 ng/mL M-CSF at 37°C in a humidified 5% CO_2_ atmosphere.

Mouse kidney proximal tubule cells isolated from NEMO^fl/fl^ or PT NEMO–null mice were treated with human rPAD4 (10 μg/mL for 6 hours) or with recombinant murine TNF-α (10 ng/mL for 6 hours) or with LPS (10 μg/mL for 6 hours) when confluent and proinflammatory cytokine induction were measured (TNF-α, MCP-1, and MIP-2). Bone marrow–derived macrophages isolated from NEMO^fl/fl^ or PT NEMO–null mice were treated with human rPAD4 (10 μg/mL for 6 hours) to determine induction of proinflammatory cytokines. We also performed p65 subunit of NF-κB immunoblotting in mouse renal proximal tubule cells treated with vehicle (saline) or with human rPAD4 (time and dose) as described (46, 56). Mouse proximal tubular cell nuclear fractions were prepared and subjected to immunoblotting with p65 subunit of NF-κB antibody from Santa Cruz Biotechnology (sc-8008) as described (13). All blots were imaged using ECL detection reagent (Thermo Fisher Scientific) and exposure using UVP Auto Chemi Darkroom and Vision Works LS acquisition and analysis software (Vision Works LS).

### Statistics.

Data were analyzed with 2-tailed Student’s *t* test, 1-way ANOVA plus Tukey’s post hoc multiple-comparisons test, or Mann-Whitney nonparametric *U* test to analyze renal injury scores. All data are expressed throughout the text as means ± SEM. *P* values of less than 0.05 were considered significant.

### Study approval.

All animal care and experimental procedures were performed under a study protocol approved by Columbia University’s IACUC.

## Author contributions

SJH and HTL conceived and designed research; SJH and MK performed experiments; SJH, MK, VD, and HTL analyzed data; SJH and HTL interpreted the results of experiments; SJH and HTL prepared figures; SJH and HTL drafted the manuscript; RMW, MK, and MSS provided material and mice; SJH, RMW, DAH, MSS, and HTL edited and revised the manuscript; and SJH, RMW, MK, DAH, VD, MSS, and HTL approved the final version of the manuscript.

## Supplementary Material

Supplemental data

## Figures and Tables

**Table 2:**
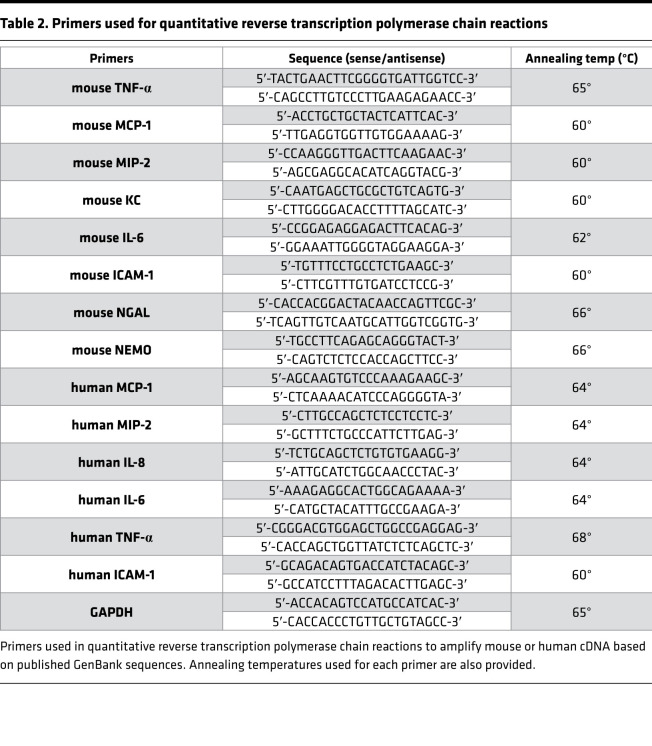
Primers used for quantitative reverse transcription polymerase chain reactions

**Table 1:**
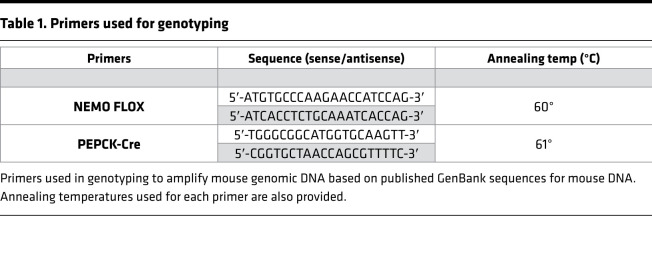
Primers used for genotyping

## Version 1 09/17/2020

In-Press Preview

## Version 2 10/02/2020

Electronic publication
