# Supplementary material for: Renal proximal tubular NEMO plays a critical role in ischemic acute kidney injury
Source: JCI Insight. 2020 Sep 17;5(19):e139246. doi: 10.1172/jci.insight.139246 (PMC7566738; doi:10.1172/jci.insight.139246)
Supplement: Supplemental data [file jciinsight-5-139246-s211.pdf]

Supplemental Figure

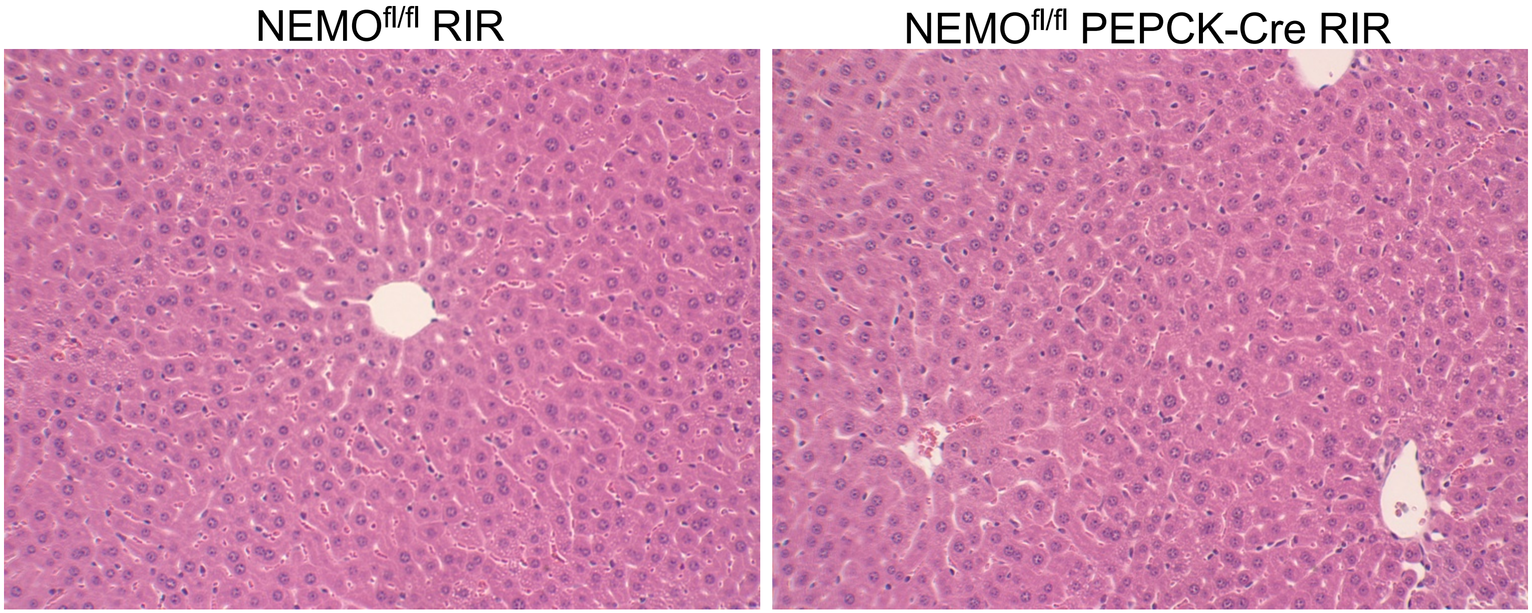

Kidney proximal tubule NEMO deletion did not alter hepatic injury after ischemic AKI. Representative H&E images (200X magnification) of livers of mice subjected to 30 min renal ischemia and 24 hrs reperfusion (representative of 5 experiments).
